# Explainable multiview framework for dissecting spatial relationships from highly multiplexed data

**DOI:** 10.1186/s13059-022-02663-5

**Published:** 2022-04-14

**Authors:** Jovan Tanevski, Ricardo Omar Ramirez Flores, Attila Gabor, Denis Schapiro, Julio Saez-Rodriguez

**Affiliations:** 1 Institute for Computational Biomedicine, Faculty of Medicine, Heidelberg University and Heidelberg University Hospital, Heidelberg, Germany; 2 Department of Knowledge Technologies, Jožef Stefan Institute, Ljubljana, Slovenia; 3 Laboratory of Systems Pharmacology, Harvard Medical School, Boston, MA USA; 4 Klarman Cell Observatory, Broad Institute of MIT and Harvard, Cambridge, MA USA; 5 Institute of Pathology, Faculty of Medicine, Heidelberg University and Heidelberg University Hospital, Heidelberg, Germany; 6 Joint Research Centre for Computational Biomedicine (JRC-COMBINE), Faculty of Medicine, RWTH Aachen University, Aachen, Germany

**Keywords:** Spatial omics, Multiplexed data, Machine learning, Intercellular signaling

## Abstract

The advancement of highly multiplexed spatial technologies requires scalable methods that can leverage spatial information. We present MISTy, a flexible, scalable, and explainable machine learning framework for extracting relationships from any spatial omics data, from dozens to thousands of measured markers. MISTy builds multiple views focusing on different spatial or functional contexts to dissect different effects. We evaluated MISTy on in silico and breast cancer datasets measured by imaging mass cytometry and spatial transcriptomics. We estimated structural and functional interactions coming from different spatial contexts in breast cancer and demonstrated how to relate MISTy’s results to clinical features.

**Supplementary Information:**

The online version contains supplementary material available at 10.1186/s13059-022-02663-5.

## Background

Highly multiplexed, spatially resolved data is becoming available at an increasing pace thanks to recent and ongoing technical developments. In contrast to dissociated single-cell data, this data informs us on the cell-to-cell heterogeneity in tissue slices while conserving the arrangement of cells [1]. Therefore, each cell can be studied in its microenvironment. We can observe the spatial distribution of the expression of markers of interest, their interactions within the local cellular niche and at the level of tissue structure. All these aspects provide an excellent platform to gain better insight into multicellular processes, in particular cell-cell communication.

The proliferation of spatial technologies leads to the generation of large amounts of data. Different technologies allow for measuring different types of molecules with varying resolution, capturing different areas of tissue with diverse numbers of readouts. Immunofluorescence-based methods allow detection of the expression of tens to hundreds of proteins at subcellular resolution [2–4] and hundreds to potentially thousands of RNA species at single-cell resolution [5]. Mass spectrometry-assisted methods enable detection of the expression of a high number of proteins at the resolution of tissue patches [6, 7] and tens of markers at subcellular resolution [8, 9], and over hundred metabolites at cellular and subcellular resolutions [10, 11]. Finally, barcoding-based approaches [12] facilitate the measurement of genome-wide expression at a resolution of hundreds of microns, i.e., several cells, and are being further developed to increase the resolution to below ten microns [13, 14]. Complementally, we are also witnessing the rapid development of methods for spatial localization that combine limited amounts of spatially resolved data with richer, but dissociated single-cell data [15–19], which can alleviate the various shortcomings of the technologies. Therefore, there is a need for methods to analyze large amounts of rich and spatially resolved data in order to discover patterns of expression, interaction, and cell functions. In fact, this has been identified as one of the grand challenges in single-cell data science [20]. These methods should ideally be able to handle the variety of produced data and scale well with future technology improvements.

Currently, there is a limited number of computational methods available for the analysis of high-resolution spatially resolved data [21]. One group of methods focuses on the analysis of the significant patterns and the variability of expression of individual markers [22–25] to describe the landscape of expression within a tissue. Another group of methods considers, more broadly, the analysis of the interactions between the markers within different spatial contexts, that is the expression in the directly neighboring cells or the effect of the expression of a marker in the broader tissue structure. The methods within the latter group focus mainly on identifying interactions in the local cellular niche, by establishing the statistical significance of the distribution of automatically identified cell types in the neighborhood of each cell [26–31]. These methods assume a fixed form of nonlinear relationship between markers or have a predefined set of spatial contexts which can be explored. Spatial variance component analysis (SVCA) [32], for example, goes a step further by examining contributions of different spatial context to the expression of markers by decomposing the source of variation to three fixed spatial contexts: intrinsic, environmental, and intercellular effects.

We introduce here a Multiview Intercellular SpaTial modeling framework (MISTy), an explainable machine learning framework for knowledge extraction and analysis of highly multiplexed, spatially resolved data. MISTy facilitates an in-depth understanding of marker interactions by profiling the intra- and intercellular relationships. MISTy is a flexible framework able to build models to describe the different spatial contexts, that is, the types of relationship among the observed expressions of the markers, such as intracellular regulation or paracrine regulation. For each of these contexts, MISTy builds a component in the model, called a view. MISTy allows for a hypothesis-driven and flexible definition and composition of views that fit the application of interest. The views can also capture functional relationships, such as pathway activities and crosstalk, cell-type-specific relationships, or focus on relations between different anatomical regions. Each MISTy view is considered as a potential source of variability in the measured marker expressions. Each view is then analyzed for its contribution to the total expression of each marker. The measured contribution points to the relevance of a potential source of interactions coming from the different spatial contexts and is estimated from the view-specific models. Our approach is modular, easily parallelizable, and thus scalable to samples with millions of cells and thousands of measured markers.

While inspired by other approaches [22–25, 26–28] to explicitly model the spatial component of the data, MISTy’s approach is unique: First, it models the complete measured expression profile and interactions instead of analyzing spatial patterns of single markers. Second, it is not limited to fixed predefined sources of variation, aggregation, or representation of the data, but allows for the flexible construction of models to analyze spatial data. Third, it does not require to annotate the cell type, state, or any other feature of the spatial unit (cell or spot). We show a more detailed comparison of MISTy with related methods in Additional file 1: Table S1.

Therefore, MISTy is not directly comparable to existing related methods. MISTy does not consider the expression of markers or their patterns individually. MISTy takes into account simultaneously the entire expression profile coming from different spatial or functional contexts assumed to explain the overall expression, as described by the modeled views. In principle, MISTy does not require annotation of cells or any other external information to describe the influence of the local niche (immediate neighborhood) or the broader tissue structure. Instead, it is agnostic to potential sources of bias and operates at the level of the available expression profile. MISTy does not assume linear or other fixed types of relationship between individual markers. Instead, it constructs a nonparametric and nonlinear model of the expression of each available marker as a function of the expression of all other markers at the same time (intrinsically) or the expression of other markers or features captured in the available views. Finally, unlike related approaches, MISTy is able to not only estimate the contribution of the available views, but also infer the importance of relations that can explain their contribution.

We validated MISTy on in silico data generated by a custom algorithm. We further applied our framework on two different imaging mass cytometry (IMC) datasets consisting of 46 and 720 breast cancer biopsies respectively. On these data sets, we demonstrated how MISTy outperforms available methods by recapitulating previous results and at the same time adding interpretation and new insights. This enabled us to discover intra- and intercellular features in triple negative breast cancer that are associated with clinical outcomes. To our knowledge, this is the first method available to connect spatially resolved single-cell measurements to the clinical outcomes without the use of cell type annotation. Finally, MISTy can extract knowledge about the interactions among signaling pathways and ligands expressed in the microenvironment from different spatial views. We demonstrate this on spatial transcriptomics data of breast cancer. These case studies illustrate the flexibility of MISTy as a framework to define exploratory and hypothesis-driven workflows for the analysis of diverse types of spatial omics data in basic and translational research.

## Results

### MISTy: Multiview intercellular spatial modeling framework

**Fig. 1:**
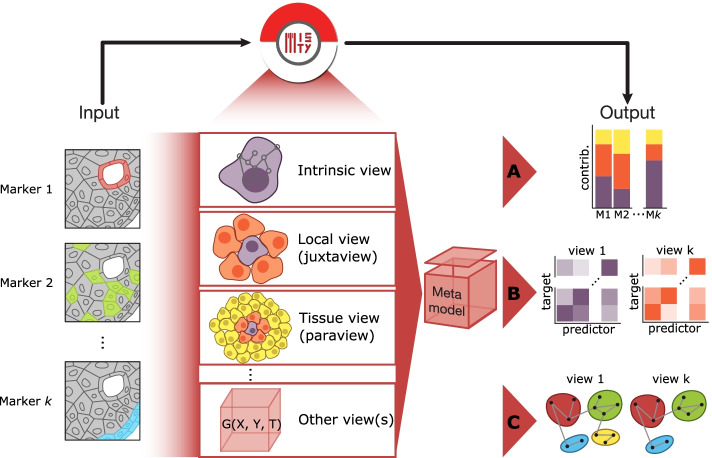
MISTy: An explainable multiview framework for modeling intercellular interactions from highly multiplexed spatial data. MISTy models marker relationships coming from different spatial views: intrinsic (intraview), local niche view (juxtaview), the broader, tissue view (paraview), or others, based directly on marker expressions or derived typology or functional characterizations of the data. At output, **A** MISTy extracts information about the contribution of different views to the expression of markers in each spatial unit. **B** MISTy also estimates the markers’ interactions coming from each view that explains those contributions. **C** These results can be described qualitatively as communities of interacting markers for each view

MISTy is a late fusion multiview framework for the construction of a domain-specific, explainable model of the expression of markers (Fig. 1, Additional file 1: Fig. S1). For each marker of interest in a sample, we can model cell-cell interactions coming from different spatial contexts as different views. For example, the first and main view, containing all markers of interest, is the intraview, where we relate the expression of other markers to a specific marker of interest within the same location. To capture the local cellular niche, we can create a view that relates the expression from the immediate neighborhood of a cell to the observed expression within that cell; we call this view a juxtaview. To capture the effect of the tissue structure, we can create a view that relates the expression of markers measured in cells within a radius around a given cell, and we call this view a paraview (see “Methods”). Importantly, MISTy is not limited to the abovementioned views. Other views can be added to the workflow that can offer insight about relations coming not only as a function of space. For example, views can focus on interactions between different cell types, interactions within specific regions of interest within a sample, or a higher-level functional organization.

Formally, we consider a matrix [*Y*]_*u*, *i*_ where each column represents a marker (*i* = 1. . *n*) and each row is a spatial location (*u* = 1. . *L*). *Y*_., *i*_is the vector made by all observations of the marker *i*. MISTy models its expression as

1 $${Y}_{\cdot, i}={\alpha}_I+{\alpha}_0{F}_0\left(\overset{\sim }{Y}\right)+{\sum}_v{\alpha}_v\Big({F}_v\left({G}_v\left(\ X,\overset{\sim }{Y},T\right)\right),$$

where $\overset{\sim }{Y}={Y}_{\cdot, \forall k\ne i}$, i.e., all markers except the target marker. *F*_*v*_ are models constructed by a machine learning algorithm for each view *v*. *G* are domain-specific functions that transform the data to generate informative variables (features) from the expression *Y* at the corresponding spatial localization *X*. Optionally, *G* can depend on other specific properties *T*, such as prior knowledge expressed as annotated functions, regions, or cell types. The *G* functions can be used to generate alternative views that can be inputs to the model function *F*. Finally, *α* are the late fusion parameters of the meta-model that balances the contribution of each view to the prediction.

MISTy always models a fixed, intraview ${F}_0\left(\overset{\sim }{Y}\right)$ as a baseline view that is independent of the spatial localization of the cell. Recall that the intraview is modeling the expression of a target marker as a function of the expression of all other markers in the same location. It is biologically expected that this intraview will be able to capture most of the variance of the expression of the measurements: the effects on the measured markers from outside of the cell are normally lower than the effects of the interactions and regulation coming from within the cell itself [33]. By design, our focus is to distinguish the non-intrinsic effects from the intrinsic baseline and estimate important interactions that supplement the explanation of the overall expression. To this end, other intercellular views are then added to ${F}_0\left(\overset{\sim }{Y}\right)$. The user can add a number *v* of additional, intercellular views and separate the effect of each view for each marker on the improvement in the predictive performance of the multiview model. We use the improvement in the predictive performance of the models as a proxy to estimate their potential as sources of interactions that can be further explored by extracting feature importances, as outlined in the following. The contribution of each view is captured by the late fusion parameters *α* of the meta-model. The intercept on the other hand captures (implicitly) the environmental effects on the mean expression of the targets, specific to the analyzed slide. For determining the contribution of the views, the fusion parameters (except for the intercept) are normalized such that they sum up to one $\overset{\sim }{\alpha_v}=\frac{\alpha_v}{\sum_{i=0}^V{\alpha}_i}$.

The above model is trained in two steps. First, the models for each view are trained independently. Second, we estimate *α* parameters of the meta-model after training the view-specific models independently, by regularized linear regression (ridge regression), to address potential issues of multicollinearity of the view-specific model predictions. The regularization parameter is determined automatically [34]. The performance of the meta-model is estimated by a 10-fold cross validation.

In terms of the choice of algorithm for training models, MISTy is a general framework and can construct models for the functions *F* with any algorithm that fulfills two requirements. First, the algorithm should construct ensemble models, with constituents trained on a bootstrap sample (bag) from the data. Second, they should be or consist of explainable models. The first criterion guarantees the unbiased use of the measurements in both steps of model training. The predictions of the constituents of an ensemble model can be made on portions of the data (out-of-bag) that were not used for their training. The second criterion means that a global explanation of the model or the feature importances can be obtained post hoc from the trained models. As proof of principle, in the implementation used in this manuscript, we consider *F* to be Random Forest [35] with 100 full, unpruned decision trees with (rounded) square root of the number of variables selected at every split. Random forests are well known, robust, and flexible models fitting the two criteria outlined above and have been shown to achieve good performance in various application areas. The feature importances for Random Forest models can be explained by the total reduction of variance achieved as the result of splitting by each of the variables in all constituent trees.

At the first level, the meta-model can be interpreted in two different ways. First, to answer the question of how much the intercellular views improve the prediction of the expression in addition to the intracellular view. This can be achieved by comparing the predictive performance of a single intracellular view vs all views combined in a meta-model. Second, by comparing the values of the fusion parameters, we can investigate how much the individual views contribute to explaining the marker expression that led to the aforementioned improvement in predictive performance (Fig. 1A).

At a second level, given this information, we can further analyze the feature importances. For each target marker, we can inspect each view-specific model and analyze how important is the contribution of each marker in that view to the prediction of the expression of the target marker (Fig. 1B). Thus, we estimate the interactions among the markers from the individual marker and view-specific models. However, for every marker, the statistical significance of the contribution of the view-specific models in the meta-model is explicitly taken into account when calculating the importances (see “Importance weighting and result aggregation”). These importances correspond to potential relationship between the predictor and the target marker in the specific spatial or other context modeled by the corresponding view. MISTy outputs the estimated importances of significant marker relations. Since these relations are based on the importance of a marker in predicting the target, they cannot be assumed to be directly causal nor directional. The relations between markers may occur through a network of intermediate interactions in the specific biological context, which can be further explored by enrichment of these relationships using curated databases of intra- and intercellular interactions (Fig. 1C). Finally, if multiple samples are available during the analysis, the relationships from individual samples are aggregated to produce robust results (see “Methods”). By aggregation, we accentuate consistently inferred interactions from individual samples and reduce the number of false positive interactions. We show a more detailed visual overview of MISTy in Additional file 1: Fig. S1.

The interpretation of the estimated relationships (interactions) is dependent on the view composition and the biological contexts of the available markers. As we show in this paper, MISTy can capture (i) a structural relationship, such as the spatial organization of cell types based on cell type identities or the expression of cells based on cell type markers, and (ii) a functional relationship between the markers, such as aspects of regulatory programs or communication-driven interactions. MISTy is designed as a method for efficient data exploration and robust hypothesis generation.

### In silico performance

#### Recovering structural relationships in in silico generated tissues

**Fig. 2:**
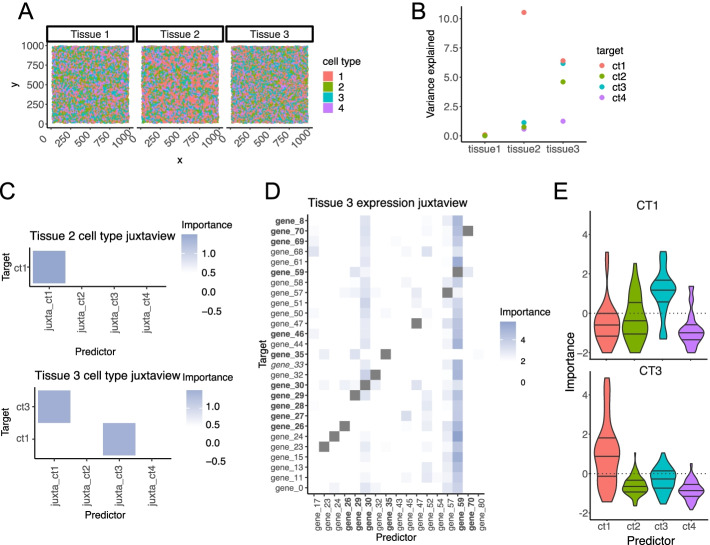
Recovery of structural relationships in the in silico tissues. **A** Voronoi diagram representation of the generated in silico tissue structures: Tissue 1—random structure, Tissue 2—structure with self-preference of a single cell type and Tissue 3—structure with mutual preferences of two cell types. **B** Amount of variance explained (percentage points) of the identity of the cell type when taking into account the information about the distribution of cell types in the immediate neighborhood. **C** Estimated importance of the relationships from the cell type distribution in the immediate neighborhood. Tissue 1 is missing as there was no information captured about the structure of the random tissue. Some of the target cell types are missing as the heatmaps contain only those targets with gain of variance explained of more than 5% and importances larger than 1 (one standard deviation above the mean importance of all predictors for that target). **D** Estimated importances of the expression of genes in Tissue 3 coming from the immediate neighborhood as predictors of the expression of the target gene. Shown are target genes with variance explained above 4%, highlighted are the interaction with estimated importance of 2 and higher. The genes shown in bold are either top 10 markers of ct0 or ct2. There is only one gene (gene_33) that is in the top 10 markers of the other two cell types. **E** Distributions of the estimated importance of the predictor-target relationship from the juxtaview of the top 10 markers of the individual cell types to the markers of cell types 1 (above) and 3 (below) in Tissue 3

We first assess the ability of MISTy to recover purely structural relationships decoupled from the influence of functional relationships. To this end, we generated three in silico tissues with specified spatial interactions between four cell types [36]. The number of cells belonging to each of the cell types is approximately equal, to remove the potential confounding effect of abundance. Cells in the generated in silico tissues are arranged in space such that the different cell types exhibit different patterns (Fig. 2A). In Tissue 1, the cell types do not show any preference to any other cell type, i.e., they are arranged randomly. This is a control tissue, where MISTy is expected to find no relationships from the spatial context. In Tissue 2, cell type 1 (ct1) exhibits preference to co-localize with itself (self-preference), while the other cell types do not have any preferences. In Tissue 3, cell type 1 and cell type 3 show mutual preference, while the other cell types have no preferences.

For each spatial pattern generated, we simulated 100 gene expression markers to create a synthetic dataset (Methods). Of the 100 markers, the distributions of 75 markers were distinguishable between cell types (“informative markers”). The simulated expression of the remaining 25 markers did not differ between cell types (“uninformative markers”). To our knowledge, this represents the most comprehensive in silico tissue model to simulate spatial interactions with continuous cell type markers.

This model only considers cell type preferences in the immediate neighborhood. Accordingly, we created view compositions in the MISTy workflows consisting of intraview and juxtaview only. The juxtaview threshold was set to the 75th percentile of all Euclidean distances between first neighbors to simulate potential errors of determining the correct threshold when applied to real data.

We considered two types of workflows: (i) The first workflow uses only information about the cell type identity and focuses on reconstructing directly the cell type composition of the tissue. (ii) The second workflow uses only information about the expression of the cell type markers and focuses on reconstructing the composition of the tissue based on the interaction of gene markers without the information about the actual cell types.

For the first workflow, the identity of the cells is captured in the intraview by one-hot encoding. In particular, each cell is described by a vector of length of the total number of cell types (4), where all values are equal to zero except for the value of the variable representing the type that the cell belongs to, which is set to one. The juxtaview then captures the distribution (total number) of the different cell types in the immediate neighborhood of each cell. For the second workflow, the intraview for each cell is represented by the expression of the 100 marker genes. The juxtaview in this workflow captures the total expression of all marker genes in the immediate neighborhood of each cell.

By modeling the interactions coming from the intraview, we would capture trivial results of prediction of identity by exclusion or co-occurrence of a large number of unique cell-type markers. While the target expressions to be modeled remain in the intraview, to avoid the aforementioned issues and to allow for modeling of self-preferences, we excluded the intraview-specific model from the meta-model in these workflows. As a result, the baseline to compare the multiview model is a model with an intercept only, i.e., a model that always predicts the mean value of the target variable.

When we applied the first workflow, since the structure of Tissue 1 is random, MISTy did not capture any information (Fig. 2B). For Tissue 2 and Tissue 3, we observed noticeable increase of variance explained for ct1 and the pair ct1 and ct3, respectively. The estimated interactions for Tissues 2 and 3 (Fig. 2C) uncover the true preferences in the tissue structure.

When we applied the second workflow to Tissue 3, we obtained the estimated interactions of the markers with high importance in the immediate neighborhood as captured by the juxtaview (Fig. 2D). For each cell type, we first ordered the gene markers by the absolute value of the difference in the mean of expression to other cell types (differentially expressed markers). We took the top 10 markers for each cell type as representative markers of that cell type. We compared the distribution of the importances of the representative markers per cell type as predictors of the representative markers of a target cell type. The mutual preference of cell types 1 and 3 is captured unambiguously by the distribution of the importances of their respective markers as predictors with significant importance (Fig. 2E).

In summary, both workflows converge on the same results—interaction between cell types 1 and 3—yet the second workflow provides a much more detailed view of the individual markers involved with the caveat of added complexity. With the two workflows, we demonstrated that MISTy is able to reconstruct the structural relationships based on annotated cell types and by the expression of cell type marker genes independently.

Of note, in a workflow similar to the first one several related approaches, for example univariate spatial pattern identification and immediate neighborhood analysis methods, would be able to capture the self-preference structure of Tissue 2. Only the latter group of methods would be able to capture the true structure of Tissue 3. In a workflow similar to the second one, only immediate neighborhood analysis methods would be able to detect marker interactions. Some of them would require preprocessing of the data (such as clustering) before they can be applied. In the following, we introduce an in silico experiment that these groups of methods cannot be applied to.

#### Recovering functional relationships from in silico generated mechanistic interaction networks

**Fig. 3:**
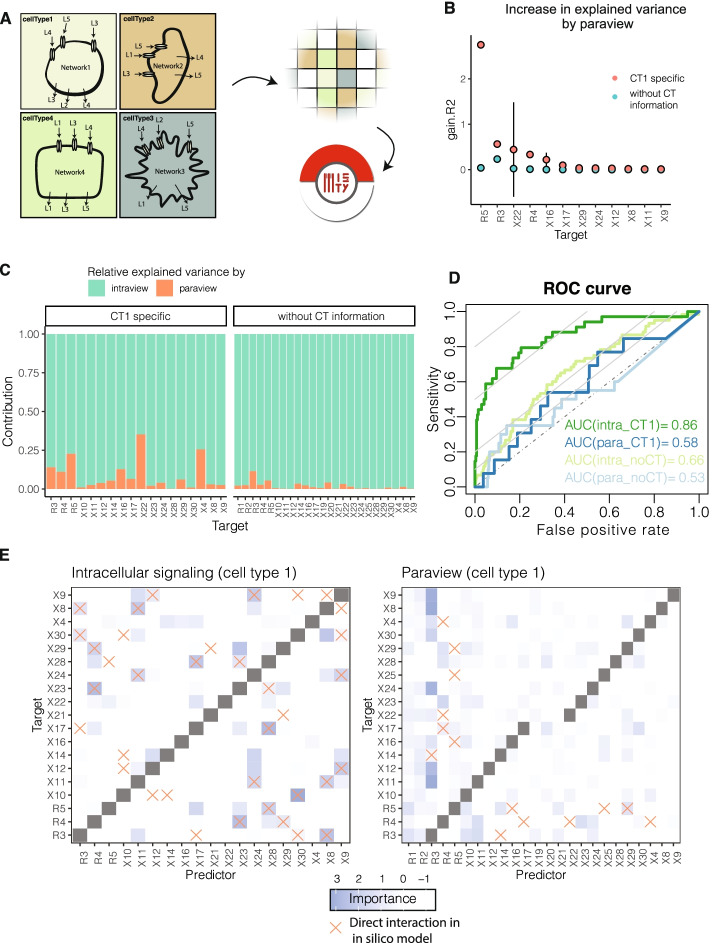
Evaluating MISTy on mechanistic in silico data. **A** MISTy was evaluated on the task of reconstruction of simulated interaction networks. Models of intra- and intercellular interactions of four different cell types (cell type specific intracellular networks are shown in Sup. Fig. 2), arranged on a grid representing a tissue, were used to simulate measurements of 29 molecular species. We considered two pipelines, (1) in which cell type information is available and (2) where cell types are not considered. **B** Increase in explained variance by adding the paraview contribution to the intraview model. Only variables with positive paraview contribution are shown. **C** Contribution of each view to the prediction of the marker expressions in the meta-model. The stacked barplot represents normalized values of the fusion coefficients of the respective views for each marker. **D** Receiver operating characteristic (ROC) depicting the aggregate performance of MISTy on the samples for the intraview and paraview, for the two cases with and without cell type information. The dashed lines represent the expected performance of an uninformed classifier, the gray iso-lines represent points in ROC space with informedness (Youden’s J statistic) equal to 0.1, 0.2, 0.5, and 0.8. **E** Predicted importance of the interactions for the intraview and paraview models for the case with cell type information (for cell type 1) together with the direct interactions from the in silico model (red crosses). Some targets had very low variance and therefore filtered out

We next assessed the performance of MISTy to reconstruct functional relationships in in silico intra- and intercellular interaction networks, decoupled from the influence of structural relationships. To estimate the robustness of MISTy to infer mechanistic molecular interactions, we created a tissue simulator that can mimic the interactions of different cell types through ligand binding and subsequent signaling events (Fig. 3A; see “Methods”) and simulated two tissue samples. The dynamic model simulates the production, diffusion, degradation, and interactions of 29 molecular species including 5 ligands, 5 receptors, and 19 intracellular signaling proteins (see Methods). The model considers four cell types (Additional file 1: Fig. S2) arranged on a two-dimensional lattice, where ligands diffuse and activate cells. The simulated values for every molecular species (except ligands) at every location are recorded and these images are passed as input to MISTy (Additional file 1: Fig. S3).

We compared two scenarios, one with no information on the cell types: in this case all the measured cells are treated equivalently; and another scenario where cell type information is considered: in this case a MISTy model is built for each cell type. The MISTy workflow consists of two views, intracellular view and broader tissue structure view (paraview). The intraview for each cell is represented by the expression of the molecular species. The paraview captures the weighted expression of all molecular species in the broader tissue structure with radius of significance of 10.

Overall, the intraview alone (Additional file 1: Fig. S4) explains a large amount of variance of the nodes that appear only in the intracellular space and that are expressed and regulated by other intracellular nodes. The paraview module increases the model accuracy mostly for receptors, which are activated by diffusing molecules in the intercellular space (Fig. 3B). For example, the increase in explained variance was the largest for R3, R4, and R5, which are the receptors that are expressed in cell type 1 (Sup. Fig. 2). We obtained similar results for all other cell types (Sup. Fig. 3 and Sup. Fig. 4). When we compare the predictive performance of this model to a model with a single intraview, we see the highest improvement in predictive performance for the expressed receptors (R3, R4, R5) in cell type 1 (Fig. 3C). Markers that were not affected by environmental interactions showed, as expected, no improvement in the paraview. It is also clear that when cell type information is considered, the model explains more variance of the targets (Fig. 3B) and the paraview contributions are generally higher (Fig. 3C).

MISTy derives an importance score for each pair of markers (see “Methods”). Using this score, we can infer intracellular and intercellular molecular interactions. To test this, we evaluated the performance of MISTy to recover interactions among markers. First, we defined the ground truth interactions from the in silico model. The 24 molecular species in the model give rise to 552 potentially interacting pairs. We considered an intracellular interaction correct if there is a direct interaction between the markers in the in silico model’s networks (Sup. Fig. 2). Further, an intercellular interaction is correct between two markers, if one marker is directly responsible for a ligand production and the other marker is activated by the same ligand. For example, X14 produces ligand L1 and L1 is activating receptor R1, thus X14 -> R1 is considered as real intercellular interaction. Between the two samples, we observed small variance in MISTy’s performance, in both the area under the receiver operating characteristic curve (AUROC) and the area under the precision-recall curve (AUPRC) (Additional file 1: Fig. S5). We aggregated the results from both samples (see “Methods” section for details) and calculated the performance for cell type 1 (Fig. 3D) and all other cell types (Additional file 1: Fig. S5). The average AUROC across the four cell types are 0.851 and 0.715 for the intrinsic and paraview, which strongly exceeds the performance of a random classifier (AUROC_random_ of 0.5). Further, the method also outperformed random classifier with respect to the AUPRC: the obtained AUPRC for the four cell types ranged between 0.581 and 0.737 for the intraview (number of true interactions 34–40; AUPRC_intra,random_ 0.062–0.065) and between 0.022 and 0.053 for the paraview (number of true interactions 10–16; AUPRC_para,random_ 0.018–0.025). The low AUPRC baseline is due to the sparsity of true intercellular interactions in contrast to the total number of interactions between the cells. The sparsity of these interactions, which is also inherent in real biological systems, adds high complexity to the task of reconstruction of the direct connections. In summary, MISTy is able to reliably extract interactions in the in silico case study.

In particular, MISTy accurately captured the downstream intracellular signaling cascades of receptors (Fig. 3E, left). Most of the false positive interactions are the results of inferring indirect or higher-order interactions, while false negative hits are likely because of the lack of perturbation: for example, node X10 in cell type 1 has no incoming edge (Supp Fig. 2), which results in a slowly decaying value in simulation. Finding the interaction partner of these types of nodes would be challenging or rather impossible for any data-driven inference method. Finding the mechanistic intercellular interactions is particularly challenging because we are looking for 10–16 real interactions among the 552 possible interactions. Most of the interactions found by MISTy correctly involve receptors (Fig. 3E, right); however, we found higher false positive rates.

With these workflows, we demonstrated the extent MISTy is able to reconstruct the mechanistic relationships in general and focused on a cell type of interest in a complex and fully observable. These results also outline the limitations of the approach, such as those caused by the presence of confounders and indirect interactions, whose effect becomes more prominent when reconstructing mechanistic intercellular relationships.

Unfortunately, the performance cannot be directly compared to other related approaches, due to their limitation to infer interactions without additional sources of information. The comparison with the most related approach, SVCA, is limited and at best only qualitative at the level of estimated contributions of the fixed views provided by SVCA. SVCA does not provide information about the potential interactions that explain the estimated contributions. Note also that the computational resources needed to construct models even on the in silico data by SVCA are orders of magnitude larger than those needed by MISTy. For a single layout with 4000 locations, on a standard configuration using 4 processor cores, SVCA took 24 h of computation time and 2.5 GB of memory, while MISTy took 18 s of computation time (4800 fold decrease) and 900 MB of memory (2.5 fold decrease).

### Application to imaging mass cytometry breast cancer datasets

#### Analyzing the importance of the tissue structure

As a first real-data case study, we applied MISTy to an Imaging Mass Cytometry dataset consisting of 46 samples of breast cancer across three tumor grades coming from 26 patients, with measurements of 26 protein markers [28]. We processed each sample independently, with 944 cells on average per sample, or 43,434 single cells in total. We designed the exploratory MISTy workflow for this task to include three different views capturing different spatial contexts and providing a foundation for comparison with SVCA: In addition to the intraview, we considered creating views by aggregating the available spatial and expression information in two ways. We created a view that describes the local cellular niche (juxtaview) and a view that describes the broader tissue structure (paraview). In order to avoid ambiguity, we set the zone of indifference for the paraview to the cutoff threshold for the juxtaview. In this way, there is no overlap in the information captured by the juxtaview and the paraview. The following results illustrate the importance of the various sources of spatial information and how MISTy can recapitulate previous findings without the need for single-cell clustering and cell type annotation using prior knowledge [28].

**Fig. 4:**
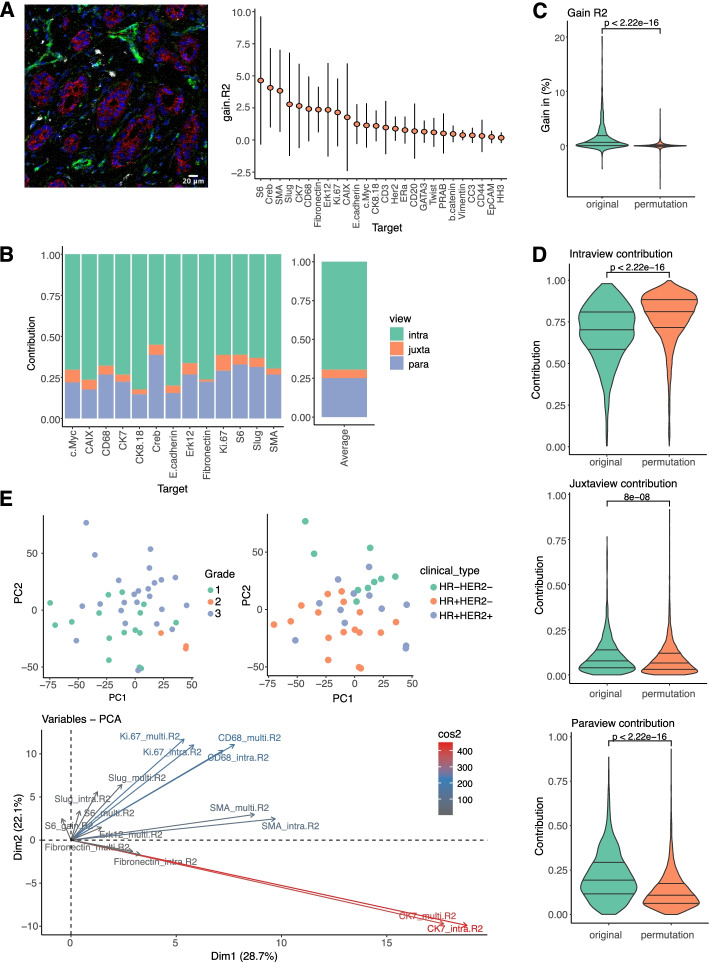
R^2^ signature and permutation analysis of IMC data from 46 breast cancer samples. **A** Imaging mass cytometry example image from a breast cancer sample (HH3 in blue, CD68 in gray, E. cadherin in red and Vimentin in green) and improvement in the predictive performance (variance explained) for all samples when considering multiple views in contrast to a single, intraview (in absolute percentage points). **B** The relative contribution of each view to the prediction of the expression of the markers. **C** Distribution of improvement in variance explained when considering multiple views in contrast to a single intraview across all markers and samples with original cell locations and 10 random permutations. The *p*-value is calculated by a one-sided Wilcoxon rank-sum test. **D** Distribution of the relative contribution of the intraview, juxtaview, and the paraview to the prediction of the markers across all markers and samples with original cell locations and 10 random permutations. The *p*-values are calculated by a one-sided Wilcoxon rank-sum test. **E** First two principal components of the R^2^ signature of the samples colored by grade and clinical subtype, and the importance of the variables of the signature in the principal component analysis. The naming of the variables is in the form Marker_Measure. The measures taken into account are variance explained by the intraview only (intra. R2), total variance explained by the multiview model (multi. R2), and the gain in variance explained (gain. R2)

We aggregated the MISTy results from all samples and we found that the multiview model resulted in significant improvements in the absolute value of variance explained of up to 20.1% over using the intraview alone, which accounted for an average of 31.8% of overall variance explained across all markers (Additional file 1: Fig. S6A). This is consistent with results obtained with SVCA, on the same data [32]. The highest improvement was detected for the markers pS6 (4.63% ± 4.99), CREB (4.07% ± 3.08), and SMA (3.83% ± 3.19) (Fig. 4A). This is expected since these three markers have distinct spatial distributions: pS6 represents “active” stroma present in distinct regions of the tumor microenvironment, SMA represents smooth muscle Actin, which is expressed in ductal structures and blood vessels; and CREB is a transcription factor commonly overexpressed and activated in tumor regions. The highest change in variance explained (20.1%) in a single sample was observed for Erk12. All top ranked markers by improvement found by MISTy are consistent with the highest improvement due to environmental effect in the results of SVCA.

We next analyzed the contribution of each view to the prediction of the multiview model (Fig. 4B). With MISTy, unlike SVCA, we were able to dissect the effect of the juxtaview and paraview. We find that a significant contribution (higher value of the fusion parameter in the meta-model) comes from the paraview compared to the juxtaview. This suggests a stronger effect from the broader tissue structure than from the immediate neighbors. The mean fraction of contribution to the prediction of the intraview was 69.5%, of the juxtaview 5.3%, and of the paraview 25.1%.

To investigate the importance of tissue structure for the modeling of spatially resolved single cell data, we performed a spatial permutation-based analysis and compared the results obtained by MISTy. The coordinates of each cell in each sample were permuted 10 times. Subsequently, we ran the aforementioned MISTy workflow on the resulting 10 new datasets. The mean gain in variance explained for the permuted data across all samples and markers was 0.5% with 49.8% of values of the gain of variance explained less or equal to 0 (Fig. 4C). The availability of true tissue structure improves the performance of the mode significantly. The estimated contribution of the juxtaview and paraview for the permuted datasets was much lower than for the original dataset, and often nearly absent. In addition, there were significantly higher contributions of the intraview than for the original dataset (Fig. 4D, Additional file 1: Fig. S6B). For the permuted dataset, the mean baseline variance explained over all samples and markers by using only the intraview was found to be consistent, i.e., remained the same as for the original dataset (31.8%).

Subsequently, we analyzed our results by the spatial variance signature (R^2^ signature) of each sample. We defined the R^2^ signature of the MISTy results for each sample by concatenating the estimated values of the variance explained using only the intraview, the variance explained by the multiview model, and the gain in variance explained for each marker. Note that the signature relates only to the results produced by MISTy for each sample and can capture different aspects of them. In this case of the R^2^ signature, the performance achieved by MISTy per target for each sample. These signatures are not related to a signature composed of biological markers for the samples and thus does not provide any insights into specific marker relationships. Here, we use the R^2^ signature representation to group the samples by similarity of the results. The use of R^2^ signature allows us to compare the results of MISTy to SVCA as reported in the manuscript describing SVCA.

The maximum length of the signature vector for each sample in this dataset is 78 (26 markers × 3 measures) dimensional, when using the information for all markers. From our signature vectors and in the following analyses, we removed the results of the performance of the markers that have less than 2% of gain in variance explained. This resulted in signature vectors of length 27 (9 markers × 3 measures).

Using the first two components of the principal component analysis (PCA) of the R^2^ signature, we identified a weak but visible structure in the samples driven by the tumor-grade and clinical subtypes, which is consistent with the findings of SVCA (Fig. 4E). The two first principal components of the R^2^ signatures of MISTy captured 50.8% of the variance of the samples compared to 30% with the spatial variance signature of SVCA. Inspecting the importance of the R^2^ signature components for the PCA analysis (Fig. 4E), we observed that the structure of the results can be explained by the gain in variance explained, which points again to the relevance of the spatial component of the data. In particular, the gain for markers CD68, ki67, and SMA were found to be the highest, suggesting that proliferation, presence, or absence of CD68 and changes in vascularization in different grades and clinical subtypes are significantly affected by the change in regulation as a result of intercellular interactions. Collectively, these results support the importance of the tissue structure for the expression of proteins at the single-cell level and overall overlap with results from SVCA and the initial performed single-cell analysis.

Since the output of SVCA only includes fractional contributions of fixed views, comparison beyond this point is not possible. As shown in the following, MISTy significantly extends the scope of possible analyses to be performed on the data.

**Fig. 5:**
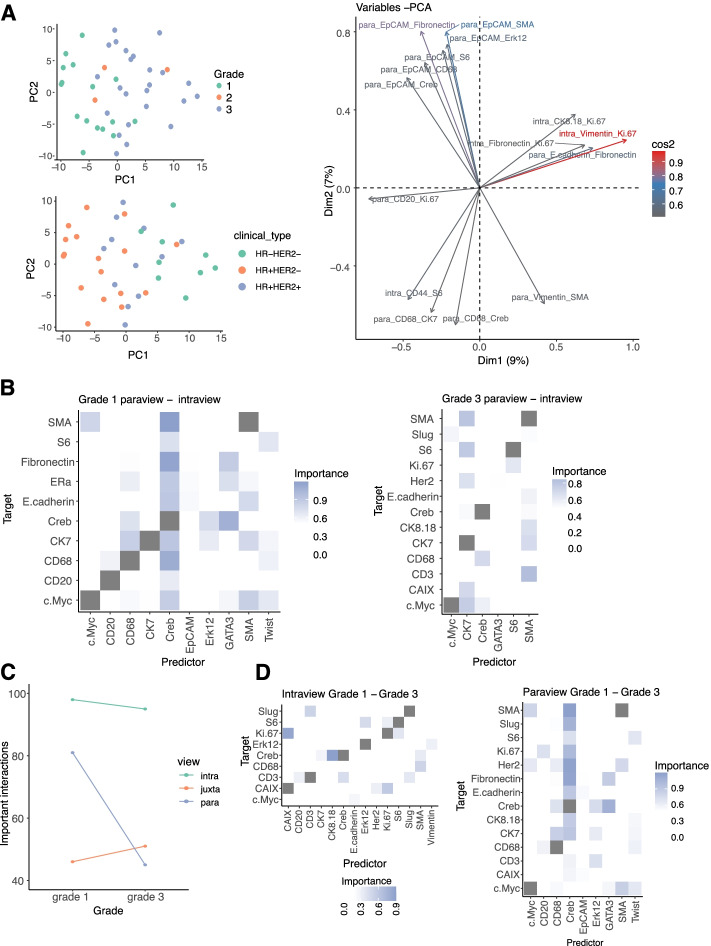
Importance signature and contrasts of IMC data from 46 breast cancer samples. **A** First two principal components of the importance signature of the samples colored by grade and clinical subtype, and importance of the variables of the signature in the principal component analysis (10 variables with the highest square cosine shown). The naming of the variables is in the form View_Predictor_Target, representing the estimated importance of the interaction between the predictor and target markers for the specific spatial context (view). **B** Intragroup contrast of importances of marker expression as predictors of the expression of each target marker between the intraview and paraview for grade 1 samples and between the intraview and paraview for grade 3 samples. **C** Change of the total number of estimated important interactions per grade (Importance ≥ 0.5). **D** Intergroup contrast of importances of marker expression as predictors of the expression of each target maker for the intraview and for the paraview between grade 1 and grade 3 samples

While the R^2^ signature allows us to analyze the differences between the samples based on predictive performance only, more insights into the relationships between the markers can be obtained by a more detailed signature at the level of the estimated importances. The importance signature is generated by concatenating the estimated importance for each predictor-target marker pair from all views. The aggregated importances are weighted by the estimated relevance of the results (see “Methods”). In this case, we created a 26-marker × 26-marker vector for 3 views (2028 dimensions). Same as before, we removed the results of the importance of the target markers that have less than 2% of gain in variance explained. This resulted in signature vectors of length 702 (9 target markers × 26 predictor markers × 3 views). The signature vector for each sample is, therefore, still large but more informative and focused on interactions. The structure in the results, driven by the tumor grade, can also be observed when visualizing the first two principal components of the importance signature (Fig. 5A). Due to richer information, they account for less (16%) of the variance of the samples compared to the R^2^ signature. By inspection of the importance of the signature components, we observed that in the two first principal components the most significant interactions that can account for the observed structure and differences among the samples come from the broader tissue view.

To confirm that the structure of the samples can be observed complementary as the result of accounting for the spatial component of the data and is not simply a result of the intrinsic expression of the markers, we performed PCA on the samples as represented by the mean expression of the markers across all cells. While the separation of the samples by grade is observable when visualizing the first two principal components (accounting for 63.9% of the sample variance), the importance of the markers that account for this separation is more uniform and different from the components of the R^2^ signature (Additional file 1: Fig. S6C). More importantly with the R^2^ and importance signatures, we were able to identify a clearer and more informative relationship between the availability of information coming from the different spatial contexts and tumor progression. This information can be then used to focus on exploratory and comparative analysis of more homogeneous groups, with lower variance of performance among samples.

#### Highlighting intergroup differences

By grouping the samples by tumor grade, we further analyzed the robust intercellular features of tumor samples. Since only a small number of samples came from grade 2 tumors, we considered only grade 1 and grade 3 tumor samples. In grade 1 samples, we observed the highest gain of variance explained for markers Cytokeratin 7 (4.92% ± 4.43), SMA (3.96% ± 3.09), and CREB (3.8% ± 2.31). In grade 3 samples, we observed the highest gain of variance explained for pS6 (6.95% ± 5.09), SMA (4.43% ± 3.39), and CREB (4.26% ± 3.52).

We further compared the aggregated results by contrasting the important interactions between the same views intragroup and intergroup. Due to the higher overall contribution of the paraview compared to the juxtaview (Fig. 4B), we analyzed the important interactions that can be extracted from the paraview model that have not been found as significant for the intraview model, i.e., capture interactions coming only from the context of the broader tissue structure. In grade 1 samples, we observed predictor markers with high importance for many target markers: the transcription factor markers CREB and GATA3, the immune cell marker CD68, and the myoepithelial marker SMA. In grade 3 samples, we observed a pronounced decline of the number of important interactions coming from the paraview compared to grade 1 samples, with the most important predictor markers being Cytokeratin 7 and SMA (Fig. 5B). This is likely representing the loss of luminal cell types (Cytokeratin 7) interacting with myoepithelial cells (SMA) in ducts and alveoli, leading to the loss of normal tissue architecture in grade 3 samples. In other words, normal tissues are highly structured, and the underlying tissue structure is critical to perform tissue relevant functions. Advanced tumors create tissue structures that are dominated by tumor cells and thus are not as dependent on cellular crosstalk and organization.

The loss of signaling during tumor progression is apparent when comparing the results view by view for the different tumor grades. The intergroup and view focused contrasts outline the interactions that were estimated as important in grade 1 samples and not important in grade 3 samples. While we observed a loss of a number of important interactions from the intraview, the loss of important interactions from the paraview is higher (Fig. 5C, D).

#### Linking estimated interactions to clinical features

To highlight the ability to associate MISTy results with clinically relevant features, we analyzed a breast cancer imaging mass cytometry dataset with outcome data, based on 415 samples from 352 patients (see “Methods” section for sample selection) [37]. As with the previous dataset, we processed each sample with MISTy independently and used the exploratory MISTy workflow with three views capturing different spatial contexts: intraview, juxtaview, and paraview.

**Fig. 6:**
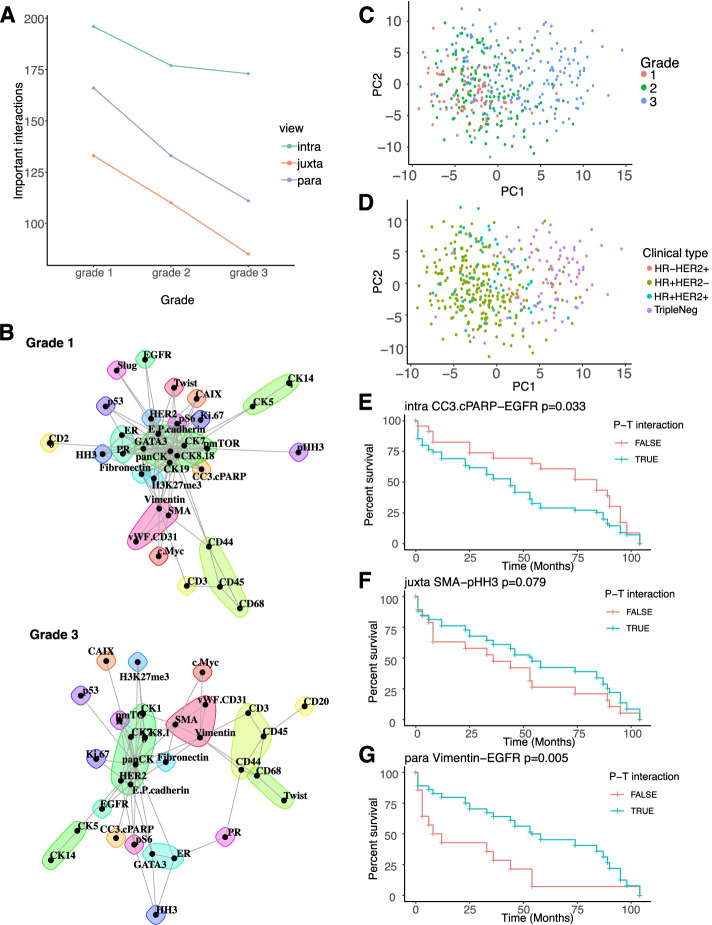
MISTy signatures can uncover clinically relevant features in IMC data from 415 breast cancer samples. **A** Change of the total number of estimated important interactions per grade (Importance ≥ 0.5). **B** Changes in the tumor microenvironment can be visualized by network community plots representing the juxtaview for tumor grades 1 and 3. For example, the green cluster represents a constant link in the juxtaview between luminal- (CK8/18+) and basal-like (CK5+) cell types across all tumor grades, while the yellow cell cluster shows an increased interaction with tumor progression of immune cells (CD68+/CD45+), B cells (CD20+) and T cells (CD3+) with cells potentially undergoing EMT (Twist+). **C** Importance signatures visualized as the first two components of a PCA highlight the separation of grade and **D** clinical subtype. Kaplan-Meier curves and *p*-values of a log rank test based on stratification by estimated importance of MISTy predictor-target interactions that were found to be correlated with the patient outcome: **E** CC3.cPARP and EGFR in the intraview; **F** SMA and pHH3 in the juxtaview, and **G** Vimentin and EGFR in the paraview

The number of important interactions, as with the previous breast cancer data set, decreased with tumor progression based on grading (Fig. 6A) across all three views. The highest median improvements were detected for similar markers as shown in the previously described breast cancer data set showing reproducibility across multiple sample cohorts (Additional file 1: Fig. S7A and B). Visualization of the network communities based on the estimated importance of the predictor—target pairs from the juxtaview for grades 1 and 3, highlights the rewiring of the tumor microenvironment during breast cancer progression. While CK14 and CK5 (green pair; top right corner) consistently interact with each other representing the basal and luminal cell compartment, immune cells seem to increase their interaction with other immune cells (e.g., B cells (CD20+)) and with cells potentially undergoing epithelial-mesenchymal-transition (EMT) (e.g., Twist+) (Fig. 6B). Next, we plotted the first two components of the PCA of the results represented by their importance signatures to visualize how tumor grade (Fig. 6C) and clinical subtypes (Fig. 6D) are distributed.

We decided to focus our further analysis on grade 3 tumors only since grade 1 and 2 samples are mostly annotated with HR+HER2− clinical subtypes (96% and 81% respectively) and the distribution of clinical subtypes for grade 3 samples is more balanced (47.6% HR−, 52.4% HR+ (out of 185), with the HR− group containing mostly triple negative subtype (79% out of 88 samples) and in the HR+ group 58,7% out of 97 samples are HR+HER2−). We next asked whether there are specific predictor-target interactions with high importance that could be linked to survival overall and for the different clinical subtypes. The performance MISTy achieved and the view contributions for the group 3 samples specifically are shown in Additional file 1: Fig. S7C and D. The predictor-target interactions that were estimated as important and are specific to the juxtaview and paraview are shown in Additional file 1: Fig. S7E and F respectively. Finally, the first two principal components of the importance signature of the samples, where we did not see further strong grouping according to clinical subtype.

To associate MISTy results with clinical outcome, we calculated the Spearman rank correlation coefficients between the estimated importance of target-marker pairs to the overall survivability in months, selecting only pairs that contain at least 30% positive importance values. Samples from patients with multiple samples were treated as independent. Next, we performed the analysis accounting for clinical subtypes by running analysis on those samples independently. In the group of HR+HER2+ samples (*n* = 8), the estimated importance of 24 predictor-target pairs (6 intraview, 12 juxtaview, 6 paraview) is significantly correlated to the overall survival (*p* < 0.05). In the group of HR+HER2− samples (*n* = 20), the estimated importance of 51 predictor-target pairs (16 intraview, 14 juxtaview, 21 paraview) is significantly correlated to the overall survival and in the group of HR-HER2+ samples (*n* = 11), the estimated importance of 39 predictor-target pairs (7 intraview, 17 juxtaview, 15 paraview) is significantly correlated to the overall survival. Importantly, we recover many interactions, without the need of single-cell annotation, that were previously shown to be linked to poor prognosis. For example, pan-cytokeratin and ER as one of the strongest correlations.

For our analysis, we focused specifically on the triple negative samples (*n* = 26), since currently no biomarkers are available that could be linked to outcome, where the estimated importance of 64 predictor-target pairs (26 intraview, 18 juxtaview, 20 paraview) is significantly correlated to the overall survival. We picked from the top predictor-target pairs correlated with overall survival for each view as an example for further analysis, but we provide all results for further experimental validation (Additional file 1: Table S2). We grouped the samples by the estimated importance of the selected predictor-target interaction. If the estimated importance for that predictor-target interaction in that sample is larger than 0.5, we consider that sample to be in the positive group; otherwise, we consider the sample to be in the negative group. We then plotted the Kaplan-Meier curves and performed a log rank test to estimate the significance of the difference in overall survivability between the two groups. We found cleaved caspase 3 and cPARP, which are both markers of cell death, when estimated to interact (predictor-target) with EGFR in the intraview, are linked to worse overall survival (Fig. 6E). In the juxtaview, we found that the absence of interaction of cells expressing myoepithelial marker SMA and pHH3, which represents cells in the cell cycle (mitosis), is linked to worse overall survival (Fig. 6F). This could hint to the importance of the distance to a blood vessel. As the last example, we also found that estimated interactions between stromal cells (Vimentin+) and cells with active RTK signaling (EGFR) to be linked to better overall survival (Fig. 6G).

In summary, we could successfully link MISTy results and signatures to clinical features and survival outcomes. The provided list of features can be used as a resource for future experimental validations, and with an increasing amount of published spatial omics datasets linked to clinical data, we expect similar studies across various disease types and experimental technologies.

### Application to a spatial transcriptomics breast cancer dataset

Key features of MISTy are that it is technology agnostic and flexible to analyze different spatially resolved data. Even more, the properties of the data obtained from different technologies can be leveraged to create different explanatory views.

To illustrate this, we analyzed the spatial gene expression profiles of two sections of a sample of invasive ductal carcinoma in breast tissue profiled with 10x Visium [38]. The 10x Visium slides contain 4992 total spots of 55 μm in diameter per captured area that enable the profiling of up to 10 cells per spot. With this technology, thousands of spatially resolved genes can be profiled simultaneously within a sample, allowing for the characterization of molecular processes.

Previously, we have shown the utility of the footprint-based method PROGENy to robustly estimate the activity of signaling pathways, in both bulk and single-cell transcriptomics [39–41]. PROGENy estimates the pathways’ activity by looking at the expression changes in downstream target genes, rather than on the genes that constitute the pathway itself. Due to the resolution and the gene coverage of 10x Visium slides, the same approach can be applied to spatial transcriptomics datasets to enhance the functional view of the data. We estimated pathway activities for two reasons: (1) to reduce the dimensions of the data of each spot into interpretable and functionally relevant features, while still using the information of as many genes as possible, and (2) to provide a set of features that are more stable than the sparse expression of marker genes.

**Fig. 7:**
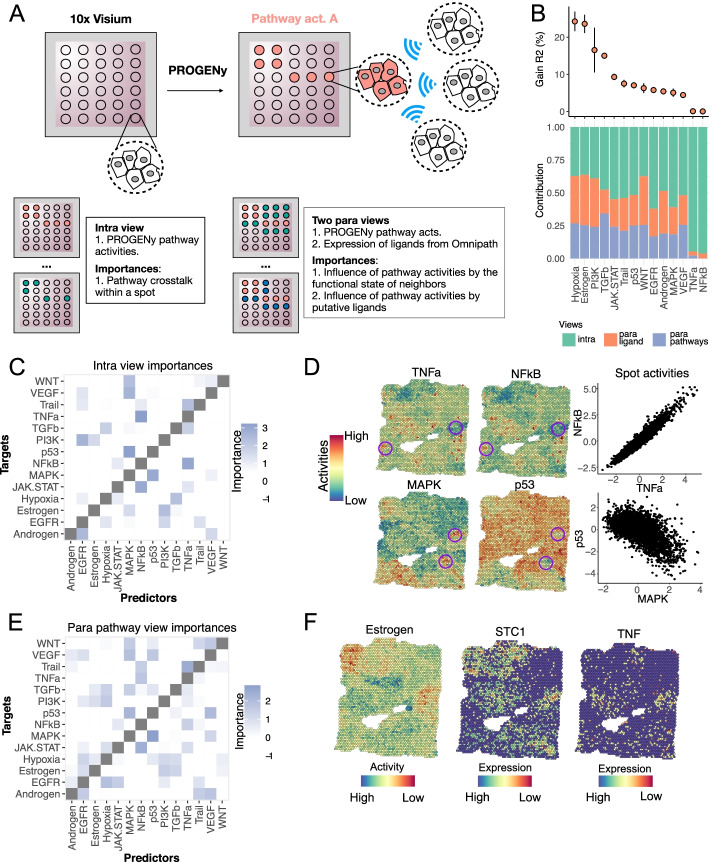
Application of MISTy to a spatial transcriptomics dataset. **A** Schematic of the MISTy pipeline used in Visium 10x slides. Each visium spot profiles the gene expression of up to 10 cells. Pathway activities were estimated with PROGENy and a MISTy model was built to predict them using two spatially contextualized views. **B** Changes in R^2^ observed in each predicted pathway after using the multiview model, reflecting the importance of the spatial context (upper panel). Contribution of each view to the prediction of the pathway activities in the meta-model. The stacked barplot represents normalized values of the fusion coefficients of the respective views for each pathway (lower panel). **C** Variable importances for the intraview. **D** Intrinsic associations of pathway activity scores of NFkB and TNFa, and p53 and MAPK. Spatial distribution of pathway activities from the first section. Circled areas exemplify niches where coordinated activities were observed. Scatterplots show the within-spot relationship between each pair of pathways. **E** Variable importances for the pathway paraview. **F** Spatial distribution of estrogen pathway activities and scaled gene expression of SCT1 (top predictor of Estrogen in the ligand paraview) and TNF

For each sample section, we estimated the activities of 14 cancer relevant signaling pathways of each spot using PROGENy [39, 41] (Fig. 7A). While pathway crosstalk mechanisms are expected within a spot, we hypothesized that the local pathway activity could also be regulated by neighboring cells in other spots to coordinate cellular processes. Therefore, we identified a set of 377 expressed genes in both sections annotated as ligands (Fig. 7A) in the meta-resource OmniPath [42] (see “Methods”) and designed a MISTy pipeline to model pathway activities using three different views: An intraview of pathway activities and two functional paraviews focusing on pathway activity a paraview using the estimated pathway activities at each patch and a paraview using the measured expressions of a set of ligands. Improvement in the prediction of pathway activities by this multiview model would provide evidence of the relevance of spatial relationships in the regulation and maintenance of the functional state of a spot. Moreover, the traceable importances of each view may suggest possible mechanisms of intercellular communication.

The multiview model improved significantly the variance explained of 12 of the 14 pathway activities (*t*-test on cross validation folds, mean adjusted *p*-value < 0.1), with improvements of up to 24% compared to the intraview model in the case of the estrogen and hypoxia pathways (Fig. 4B). We found a mean contribution of 55% of the intraview, 24% of the ligand expression paraview, and 21% of the pathway activity paraview to the prediction of pathway activities in the multiview model (Fig. 7B). We compared these results to the model performance in five iterations of slides with permuted layouts to provide further evidence of the importance of spatial information in the prediction of marker pathway activities (see “Methods,” Additional file 1: Fig. S9A,B). As expected, in these random slides, we observed no improvements in variance explained when fitting models with spatially contextualized views (Additional file 1: Fig. S9A). Moreover, when we compared the view’s contributions of the models fitted to random and original slides, lower contributions of the paraviews were recovered for the random models (Additional file 1: Fig. S9B, Wilcoxon test *log*_10_*p* <  − 10). These results confirmed that MISTy models are able to extract informative spatial relationships between markers in tissue samples where spatial organization is expected.

The importances of the features used as predictors in each view are consistent with biological processes. In the intraview (Fig. 7C), we recovered, among others, associations between NFkB and TNFa, and P53 and MAPK that have been reported previously [39]. These results capture pathway crosstalks within a spot as illustrated in the spatial distribution of pathway activities shown in Fig. 7D. Predictor importances in the pathway paraview captured similar associations as the ones captured by the pathway intraview (Fig. 7E). The paraview importances, however, reflect patterns of tissue organization in which multiple neighboring spots share similar cellular states in larger areas. If a relationship between two pathways A and B is observed within a spot and a coordinated local activity of these pathways is happening, then the activity of pathway A of the neighbors of a given spot indirectly explains its pathway B activity. For example, the obtained paraview relationship between NFkB and TNFa, and P53 and MAPK (Fig. 7E) explained the regions where a collection of spots showed coordinated higher or lower activities of these pathways (Fig. 7D, circled areas). Additionally, new associations between pathways became relevant when taking into account the functional state of the neighbors of each spot (Fig. 7E). In hypoxia, where the contribution of the para pathway view to the multiview model was 35%, estrogen, PI3K, p53, and WNT pathway activities had the highest importances, besides EGFR and TGFb that were recovered from the intraview importances too (Additional file 1: Fig. S9C). The local expression of putative ligands contributed mostly to the prediction of estrogen, WNT, and hypoxia (para ligand view contribution ≥ 34%, Fig. 7B). We annotated each ligand-pathway interaction using Omnipath. We recovered the potential target receptors of all predictor ligands and assigned them to one of the 14 pathways in PROGENy based on the whole collection of annotations stored in Omnipath. Additionally, we annotated each predictor ligand as a direct byproduct of a pathway if they belonged to one of the transcriptional footprints in PROGENy. From the 195 most important ligand-pathway interactions (importance ≥ 2), 130 could be annotated as described above. The 65 unannotated interactions could represent novel context-dependent intercellular processes and show how MISTy could be used as a hypothesis generation tool. Among the top annotated interactions observed between the pathway activities and ligands (Additional file 1: Fig. S9D), we recovered the relationship between STC1 and estrogen pathway activities (Fig. 7F). STC1 is a glycoprotein hormone that is secreted into the extracellular matrix and has been discussed in the literature as a promising molecular marker in breast cancer [43]. TNF, STC1’s reported receptor, showed similar spatial patterns in the slide (Fig. 7F), suggesting a potential intercellular mechanism that mediates estrogen pathway activity. High importances to predict estrogen pathway activity were observed for other estrogen-receptor-dependent genes such as EFNA1 and EDN1, as well as for the estrogen responsive gene TFF1 (Additional file 1: Fig. S9E). Interestingly, we observed that ligand importances clustered pathways that shared para pathway interactions, such as p53, MAPK, and TGFb. Altogether, our results showed that MISTy was able to improve the prediction of pathway activities by incorporating their spatial context. Moreover, we were able to identify known and novel spatial dependencies between pathway activities and ligands that reflect the functional organization of the tissue.

## Discussion

Here we present MISTy, an explainable framework for the analysis of highly multiplexed spatial data without the need of cell type annotation. It can scale and is technology agnostic enabling the analysis of increasingly complex data generated by recent and upcoming technologies. MISTy complements other methods that leverage spatial information to explore intercellular interactions. The current approaches focus mainly only on the local cellular niche, i.e., the expressions measured in the immediate neighborhood of each cell [26–29, 31]. Other methods that consider the broader tissue structure are relatively inflexible [30, 32]. They consider a fixed form of nonlinear relationship between markers at predefined spatial contexts (e.g., fixed distance), and they do not scale well due to their high computational complexity. In contrast, MISTy offers a flexible range of spatial analyses in a scalable framework. We present a selected set of workflows for the analysis of spatial data, using not only the marker expressions but also derived features, such as pathway activities.

We established a performance baseline for MISTy on in silico data before applying MISTy to real-world data. We showed that MISTy achieves high performance on the task of reconstructing the intra- and intercellular networks of interactions.

We then applied MISTy to three real-world spatial omics data sets from breast cancer samples. We applied MISTy on imaging mass cytometry data, capturing dozens of protein markers at (sub) cellular resolution. The results show that we were not only able to recapitulate results from the literature without prior-knowledge-based cell type annotation, but to also generate new hypotheses. Our results show that the information that is available from the expression of markers in the broader tissue structure is often more important than their expression in the local cellular niche. Of note, this result, which is biologically intuitive, could not be found with previous methods that do not distinguish between para- and juxtaview. This highlights that not only cellular niches but also the tissue structure has a direct impact on cellular states and should be included in the “microenvironment” definition. Furthermore, we show how MISTy finds interactions that are associated with clinical features.

Finally, we applied MISTy on a spatial transcriptomics data set measured with 10x Visium. Here, thousands of transcripts are measured in spots containing several cells. Given the richness of the data, we were able to go a step further and consider the analysis of functional features, in the form of pathway activities that were inferred from the data. In particular, we showed the crosstalk between pathways and the ligand-pathway interactions in the context of the broader tissue structure in breast cancer. Our results showed that MISTy in combination with functional transcriptomics tools and prior knowledge can be used in spatial transcriptomics to uncover coordinated functions that are maintained in niches of the tissue. Moreover, the explanatory component of the multiview model provides relevant predictors that could become the base of mechanistic models.

Although the interactions extracted by MISTy cannot be considered directly as causal, they can facilitate the downstream analysis of biological systems at the tissue level in several directions: (i) to predict the behavior of systems under perturbations, by using the MISTy model to generate marker expressions based on the new conditions; (ii) to guide the reconstruction of multicellular causal signaling networks, using databases to identify mechanisms giving rise to the extracted interactions; and subsequently (iii) to construct mechanistic models of the dynamical behavior of the system constrained by the extracted explanations.

The work we presented lays the foundation for further exploration of MISTy in several directions. One direction is to address the scalability of MISTy to millions of cells and thousands of markers per sample, which is beyond what the available technologies can offer, but is likely to come in the near future. To do this, we are exploring approximate but accurate methods to replace the computationally expensive step of generating views where the pairwise distances between all cells need to be calculated. Another direction is the exploration of the performance that can be achieved by MISTy with different ensemble approaches using various types of explainable constituent models. Furthermore, MISTy can be used to generate more specific views. In particular, views that capture the spatial expression of specific cell types, so that we can dissect the spatial interactions between different cell types, or views that focus on regions of the tissue, for example, healthy vs pathological, where we would model the interactions between the functionally different regions. Of special interest is also the specialization of MISTy workflows that focus on the analysis of ligand-receptor interactions while taking into account the spatial context. To this end, we look towards combining MISTy with complementary tools, such as GCGN [30], MESSI [31], cell2cell [44] and Tensor-cell2cell [45]. In particular, we plan to explore the integration of databases of intercellular signaling as modeling bias as in GCGN, focusing workflows on the communication between pairs of cell types as in MESSI. In another direction, cell2cell results can be used to inform ligand-receptor analysis with MISTy, or use MISTy’s importance signatures as the input communication score matrices for Tensor-cell2cell. Finally, MISTy generates a model for each marker of interest that can be readily used to make predictions of marker expressions under different conditions. For example, we can increase or reduce the expression of a certain marker in silico and explore the effects of the new condition.

## Conclusions

In summary, we believe that MISTy is a valuable tool to analyze spatially resolved data, adaptable to multiple data modalities and biological contexts, that will also evolve as experimental techniques improve. An implementation of MISTy as an R package named mistyR (https://saezlab.github.io/mistyR/) is fully documented and freely available from GitHub, Bioconductor, and as a Docker image.

## Methods

### In silico tissue structure

We simulated the data distribution for each cell type by sampling from a multivariate normal distribution, where each marker had a randomly chosen mean expression with a narrow variance. To create informative markers between cell types, we randomly adjust the mean of a marker for each cell type, such that the distributions of a given marker expression for each cell type are likely to be non-overlapping. For uninformative markers, mean expression is the same between cell types. After choosing marker-wise mean expression and adjusting the means for informative markers, a synthetic dataset is generated by sampling from this distribution. Cells of specific types are matched with their corresponding spatial location from the in silico tissue generation to construct a synthetic spatial dataset.

### In silico mechanistic model

The mechanistic in silico model is a two-dimensional cellular automata model that focuses on signaling events; therefore, cell growth, division, motility, and death are neglected. First, we created two random layouts. To account for cellular heterogeneity in the tissue, we assigned one of four different cell types *CT*1, …*CT*4, to each spot of the layout or left it empty (intercellular space). Each of these cell types has a distinct set of receptors expressed and distinct intracellular wiring (Sup. Fig. 2). To keep the model simple, we considered 29 biological species S = {ligands: L1-L5; receptors: R1-R5 intracellular proteins: X10-X29}. The intracellular processes involve the ligand activation of receptors and downstream signaling nodes, and ligand production/secretion (Fig. 3A). The model simulates the production, diffusion, degradation, and interactions of these 29 molecular species on a 100-by-100 grid. Ligands are produced in each cell type based on the activity level of their production nodes and then freely diffuse, degrade, or interact with other cells on the grid. Other molecular species involved in signaling are localized in the intracellular space and their activity depends on ligand binding and intracellular wiring.

The model is formally stated by the following partial differential equations for each species:

2 $$\frac{\partial {c}_s\left(x,y,t\right)}{\partial t}={d}_s\varDelta {c}_s\left(x,y,t\right)+{P}_s\left(x,y,t\right)-{D}_s\left(x,y,t\right)$$

This equation describes the diffusion, the production/activation, and the degradation of the species. We made the following assumptions: *c*_*s*_(*x*, *y*, *t*) is the concentration of species *s* ∈ *S* at the grid point (*x*, *y*) at time *t*. The diffusion is homogenous across the image, the diffusion coefficient of species *s* is *d*_*s*_. Only ligands are diffusing, and other intracellular molecules cannot leave the cell.

The production term includes the generation of ligands and the activation of intracellular proteins and receptors. Production depends on the cell types and the activity of the production node: the ligand production depends linearly on the nodes above them (supp Fig. 2): *P*_*i*_(*x*, *y*, *t*) = *α*_*i*, *ct*_*X*_*i*_(*x*, *y*, *t*) for *i* ∈ {*L*1, *L*2, *L*3, *L*4, *L*5} and *ct* ∈ *CT*, the *α*_*i*, *ct*_ coefficient defines which cell type produces which ligands and how strongly the production depends on the activity of the production node.

Ligands are specific and activate only the corresponding receptors, e.g., L1 activates R1, L2 activates R2, etc. The activation of the receptor depends on the concentration of the ligand at the location of the cell.

For intracellular proteins, the protein activity depends on the activity of upstream nodes. An interaction *X*_*i*_ *-> X*_*j*_ is translated to the equation: ${P}_j\left(x,y,t\right)={{\beta_{j,i}}^{ct}}_{c_i\left(x,y,t\right)}$, where *β*_*j*, *i*_^*ct*^ encodes the strength of interactions between the nodes in cell type *ct.*

Degradation is proportional to the concentration of ligands, intracellular proteins, and ECM, *D*_*s*_(*x*, *y*, *t*) = *γ*_*s*_*c*_*s*_(*x*, *y*, *t*), where *γ*_*s*_ is a constant degradation coefficient.

The above model was simulated from a randomized initial condition, and the activity distribution (Additional file 1: Fig. S3) was achieved. We considered all markers except the ligands as available measurements for the MISTy workflow. In contrast to the included measurements, our assumption is that the expression of ligands would be more difficult to capture in a real experiment and therefore excluded them.

We aggregated the interactions from the mechanistic model for the different cell types in joint binary matrices of directed ground truth interactions for the different views. To compare the matrices to the importance matrices from the output of MISTy, we transformed the joint matrices into undirected matrices *A*_*u*_ =  *sgn* (*A* + *A*^*T*^). We then quantified the performance of MISTy for the task of reconstruction of intra- and intercellular networks from the true and the extracted interaction matrices.

### Data acquisition and processing

#### Imaging mass cytometry

The first imaging mass cytometry dataset consists of 46 samples from 26 breast cancer patients with varying disease grades [28]. The original data consisted of 50 samples, from which we removed samples coming from normal tissue. The raw data was segmented and single cell features were extracted with histoCAT. The samples contain between 267 and 1455 cells with measured expression of 26 proteins/protein modifications. The cell-level data was preprocessed as defined in Arnol et al. [32] in order to assure the validity of direct comparison of results.

The second imaging mass cytometry dataset consists of 720 samples from 352 breast cancer patients from two cohorts, with long-term survival data available for 281 of those patients [37]. The samples contain measurements of 37 proteins/protein modifications. The raw data was segmented, and single-cell features were extracted with histoCAT. The cell-level data was preprocessed as in the original study. To ensure robustness of the results, we filtered samples containing less than 1000 cells, samples coming from a normal or control tissue, and samples without annotated tumor grade or clinical subtype, resulting in a total of 415 samples for our analysis.

#### Spatial transcriptomics

The data and sample information were obtained from 10x Genomics [38]. The data consists of spatial transcriptomic measurements of two sections of a sample analyzed with 10x Genomics Visium. The sections come from tissue from a patient with grade 2 ER^+^, PR^−^, HER2^+^, annotated with ductal carcinoma in situ, lobular carcinoma in situ, and invasive carcinoma. The mean sequencing depths were reported to be 149,800 and 137,262 reads per spot for a total of 3813 and 4015 spots per section respectively. The median UMI counts per spot were reported as 17,531 and 16,501, and the median genes per spot as 5394 and 5100 respectively. The raw data was preprocessed and count matrices were generated with *spaceranger-1.0.0*. Individual count matrices were normalized with *sctransform* implemented in Seurat 3.1.2 [46]. For each spot, we estimated signaling pathway activities with PROGENy’s model matrix using the top 1000 genes of each transcriptional footprint. We retrieved from Omnipath [42] all proteins labeled as ligands and in each dataset, we filtered all ligands whose expression was captured in at least 5% of the spots.

#### View generation

We consider a dataset *D* = [*X*_(*n* × *s*)_ *Y*_(*n* × *k*)_], represented as a matrix of dimensions *n* × (*s* + *k*) of spatially resolved highly multiplexed measurements of a sample, where *n* is the number of measured units (pixels, cells, patches) available in the sample, *s* is the number of spatial dimensions in the geometry matrix *X*, and *k*is the number of measured markers in the expression matrix *Y*.

The juxtaview was generated by summing the expressions of its direct neighboring cells, i.e., ${G}_c={\sum}_{j\in {N}_c}{\overset{\sim }{Y}}_{j,\cdot }$, where *N*_*c*_represents the set of neighboring cells of cell *c*. The neighboring cells for each cell can be determined either during image segmentation, for example by setting a threshold of membrane-to-membrane distance, or, as in the case for the application of MISTy on IMC data, by post hoc neighborhood estimation. For the application of MISTy on IMC data, the neighborhood of each cell in a sample was estimated by constructing a cell graph by 2D Delaunay triangulation followed by removal of edges with length larger than the 25th percentile of all pairwise cell distances across all samples, which corresponded to 11.236 microns from the cell centroid.

The paraview was generated by weighted aggregation of the expressions of all cells (patches) from the sample ${G}_c={\sum}_{j=1}^n1\left({d}_{cj}\ge z\right)w\left({d}_{cj},l\right)\overset{\sim }{Y_{j,.}}$, where *w* is a weighing function, *d*_*cj*_is the Euclidean distance between cells *c* and *j*, calculated from matrix *X*, *l* is a parameter controlling the shape of the weighting function *w*, and *z* is a parameter controlling the zone of indifference. A juxtaview and a paraview with no zone of indifference will both contain the expression of the markers in the neighboring cells. Considering a zone of indifference larger than the immediate neighborhood would ensure that only the juxtaview will capture the immediate neighborhood, while the paraview will capture only the broader tissue structure excluding the immediate neighborhood.

We can assume the cells that are closer affect the expression within the cell more than cells that are farther away to various degrees. The weighing function *w* controls the contribution of the expression coming from the broader tissue structure as a function of the distance and a parameter *l* that captures the radius around the cell where we consider the cells in the broader tissue structure to be significantly contributing to explaining the expression in the cell. Examples of weighting functions in MISTy are the families of radial basis $w={e}^{-\frac{d^2}{l^2}}$, exponential $w={e}^{-\frac{d}{l}}$, linear $w=1-\frac{d}{l}$, and constant functions *w* = 1(*d* ≤ *l*).

For the application of MISTy to both IMC and spatial transcriptomics data, we considered the family of radial basis functions for weighting and optimized the value for the parameter *l*. For each IMC sample, we constructed models for each marker, with parameter *l* ∈ {25, 50, 100, 200, 400}. This corresponds to an effective radius of influence of 25 to 400 pixels or micrometers. The mean values of the parameter l across all samples for all markers are shown in Additional file 1: Fig. S8. Given the resolution of 10x Visium, MISTy models for spatial transcriptomics were built for each pathway activity considering in the paraview the family of radial basis functions for weighting with values of parameter *l* ∈ {2, 5, 10}, corresponding to a radius of influence of up to 10 spots. Then, for each marker, we selected the value for *l* such that the estimated improvement in predictive performance by using the multiview model in contrast to the intraview model is maximized. For each model (Random Forest), we estimate the predictive performance by measuring the variance explained on out-of-bag samples.

#### Importance weighting and result aggregation

To calculate the interaction importances from a sample, we used information from the two layers of interpretability and explainability: the values of the fusion parameters (*α*_*v*_in Eq. (1)) in the meta-model and their respective *p*-values *p*_*k*_^(*v*)^ for each target marker *k* and the importances *I*_*kj*_^(*v*)^of features *j* for the prediction of each target marker extracted from the predictive model for view *v* yields the MISTy interaction importance:

3 $${M_{kj}}^{(v)}=\frac{{I_{kj}}^{(v)}-\bar {{I_k}^{(v)}}}{{\sigma_{{I_k}^{(v)}}}^2}\left(1-{p_k}^{(v)}\right)$$

Since the importances *I*_*kj*_^(*v*)^extracted from a Random Forest model (used for the current instance of MISTy) represent the amount of variance reduction in the target expression, the MISTy interaction importances correspond to the standardized value (by mean $\bar {{I_{kj}}^{(v)}}$ and variance ${\sigma_{{I_k}^{(v)}}}^2$) of the variance reduction weighted by the quantile 1 − *p*_*k*_^(*v*)^ of the statistic under the null hypothesis of zero contribution of the fusion coefficient for view *v* for target *k*in the linear meta-model.

MISTy is conceived to be a framework applicable to any type of omics data. The performance measures and the estimated relationships are independent from the properties of the variables used to describe the data. The machine learning models (Random Forest) trained on the specific views are invariant to the scale of the predictor or the target variables. The measure of the performance of the model (variance explained) is also chosen such that issues with scale are avoided during training and interpretation.

MISTy infers the interactions between the variables by the proxy task of predicting the expression, abundance, activity, or any other quantity of the target variables and estimating the importance of each of the predictor variables for this task. The estimated relationships/importances are related to the amount of reduction of variance and not to absolute values. The importance derived from variance reduction can be generalized to any measure of impurity or values extracted by other feature importance estimation methods, given the model constituents of MISTy. Since the MISTy importances are standardized, importances from multiple samples can then be aggregated by simple averaging, while their interpretation remains the same.

For views that contain the same set of predictors as targets, we also identified the communities of interactions from the estimated importances. For this, we transformed the square matrix *A* of estimated predictor-target interactions to an undirected graph adjacency matrix as *A*_*p*_ = *A* + *A*^*T*^. We then extract the community structure from the graph using the Louvain algorithm [47], a commonly used algorithm for community detection by grouping nodes, such that the modularity of the graph is maximized.

#### Permutation of the slides

To evaluate the performance of MISTy models in samples with no spatial organization, we generated random samples for both the IMC and spatial transcriptomics data and ran the same pipeline as for the original data. We permuted the coordinates of each spatial unit (cell or spot) for each slide ten times for the IMC data and five times for the spatial transcriptomics data. Results were grouped and compared to the ones obtained in the slides with the original spot layout.

#### Annotation of predictive ligands from the spatial transcriptomics pipeline

To facilitate the interpretation of the paraview ligand importances observed in the spatial transcriptomics models, we assigned each ligand to a PROGENy pathway in two ways: (1) as a byproduct of pathway activation or (2) as a potential activator of a pathway. A ligand was considered as a byproduct of a PROGENy pathway if it was part of its top 1000 footprint genes. A ligand was considered as a putative activator of the pathway it predicted if at least one of its possible receptors could be assigned to it. We used OmnipathR to annotate each receptor using the *import_omnipath_annotations* function and regular expressions to filter annotations associated with the PROGENy pathway of interest. Only ligands with a paraview importance ≥ 2 were considered in this annotation (Additional file 1: Fig. S9D).

## Supplementary Information

**Additional file 1. **Supplementary Figures S1 to S9 and supplementary Tables S1 and S2.

**Additional file 2.**

## Data Availability

The source code of mistyR is publicly available from Bioconductor [48]. The exact version (1.3.5) of the source code used for the manuscript is available from Zenodo [49]. The source code for the analysis of the data is available from a public repository [50]. In silico tissue generation code is available from a public repository [51]. The generated mechanistic in silico data is available from a public repository [50, 51]. The Imaging Mass Cytometry data is publicly available from Schapiro et al. [28] and Jackson, Fischer et al. [37]. The Visium spatial transcriptomics data is publicly available from 10x Genomics [38].
