# Supplementary material for: Explainable multiview framework for dissecting spatial relationships from highly multiplexed data
Source: Genome Biol. 2022 Apr 14;23:97. doi: 10.1186/s13059-022-02663-5 (PMC9011939; doi:10.1186/s13059-022-02663-5)
Supplement: Supplementary file 1 — Additional file 1. Supplementary Figures S1 to S9 and supplementary Tables S1 and S2. [file 13059_2022_2663_MOESM1_ESM.pdf]

Additional file 1

Supplementary figures and tables to the manuscript *Explainable multi-view framework for dissecting spatial relationships from highly multiplexed data*.

Figures S1 to S9.

Tables S1 and S2.

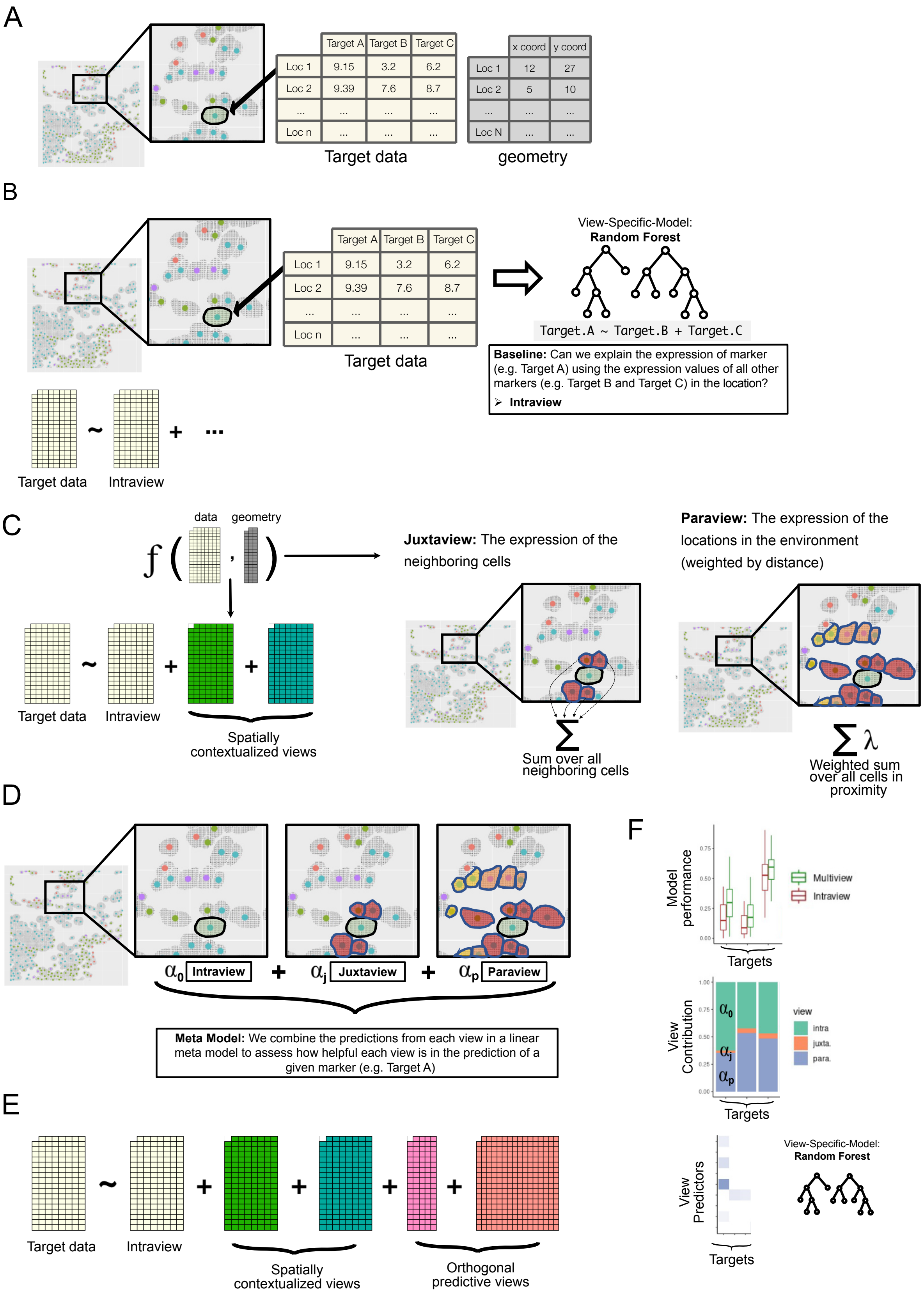
A
Target A
Target B
Target C
Loc 1
9.15
3.2
6.2
Loc 2
9.39
7.6
8.7
...
Loc n
Target data
x coord
y coord
Loc 1
12
27
Loc 2
5
10
Loc N
geometry
B
Target data
View-Specific-Model:
Random Forest
Target.A ~ Target.B + Target.C
Baseline: Can we explain the expression of marker (e.g. Target A) using the expression values of all other markers (e.g. Target B and Target C) in the location?
Intraview
~
+
...
Target data
Intraview
C
data
geometry
f
Juxtaview: The expression of the neighboring cells
Paraview: The expression of the locations in the environment (weighted by distance)
Target data
Intraview
Spatially contextualized views
Sum over all neighboring cells
Weighted sum over all cells in proximity
D
Intraview
Juxtaview
Paraview
Meta Model: We combine the predictions from each view in a linear meta model to assess how helpful each view is in the prediction of a given marker (e.g. Target A)
E
Target data
Intraview
Spatially contextualized views
Orthogonal predictive views
F
Model performance
Multiview
Intraview
Targets
View Contribution
view
intra
juxta.
para.
Targets
View Predictors
View-Specific-Model:
Random Forest
Targets

***Fig. S1*. MISTy view composition and modeling.** *(A) The input for MISTy is a matrix of marker expressions and the location of each spatial unit. (B) The intraview consists of the matrix of marker expression. The intraview specific model, relates the expression of all other markers to a target marker within the same spatial location (cell/spot/spatial unit). The model used in our work to learn these relations is Random Forest. We use the intraview as a baseline as we expect most of the information to come from the same location and we want to be able to distinguish all other relationships that come from outside of this view. (C) MISTy allows for composing views that capture different spatial contexts. For example the juxtaview captures the local cellular niche (immediate neighborhood) by the sum of the expression of all markers of the direct neighbors of the spatial unit. The paraview captures the broader tissue structure by a weighted sum of the expression of all markers in the tissue. The closer a spatial unit is, the more weight is given to the expression coming from that unit. (D) After training models for each view, i.e., relating the expression of the variables in that specific view to the expression of the target marker as given in the intraview, their predictions are combined by a linear meta model. (E) MISTy is flexible and allows for adding as many views as needed capturing different spatial contexts or orthogonal information. (F) MISTy generates three general outputs. The first gives information about the amount of information that is gained when considering multiple views in contrast to the intraview only. The second gives information about the contributions of each view, as captured by the parameters of the meta-model. The third gives an insight into the predictor-target relationships that were found to be most important for each view.*

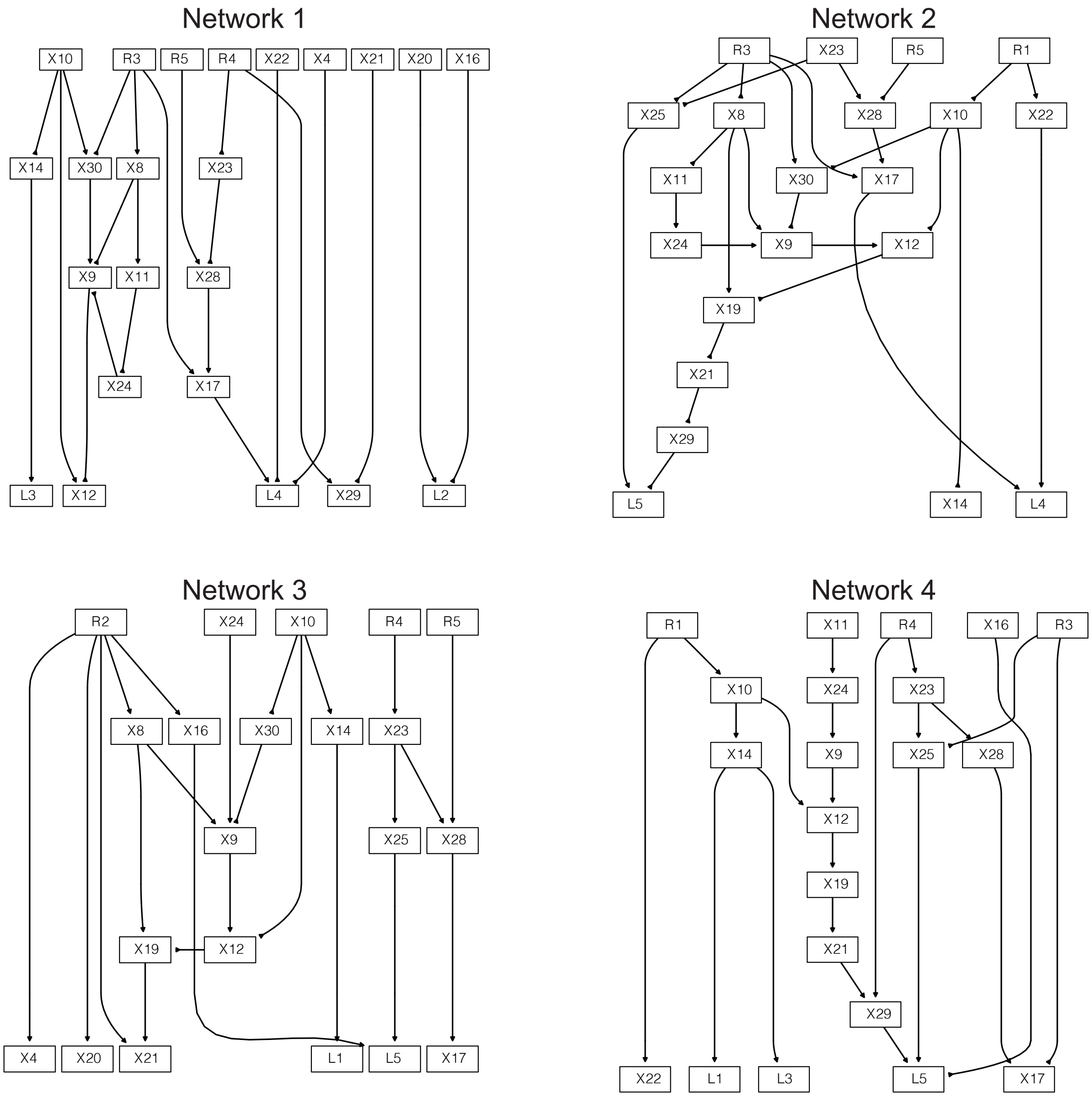

Fig. S2. Intracellular signaling networks used in the mechanistic in silico models.

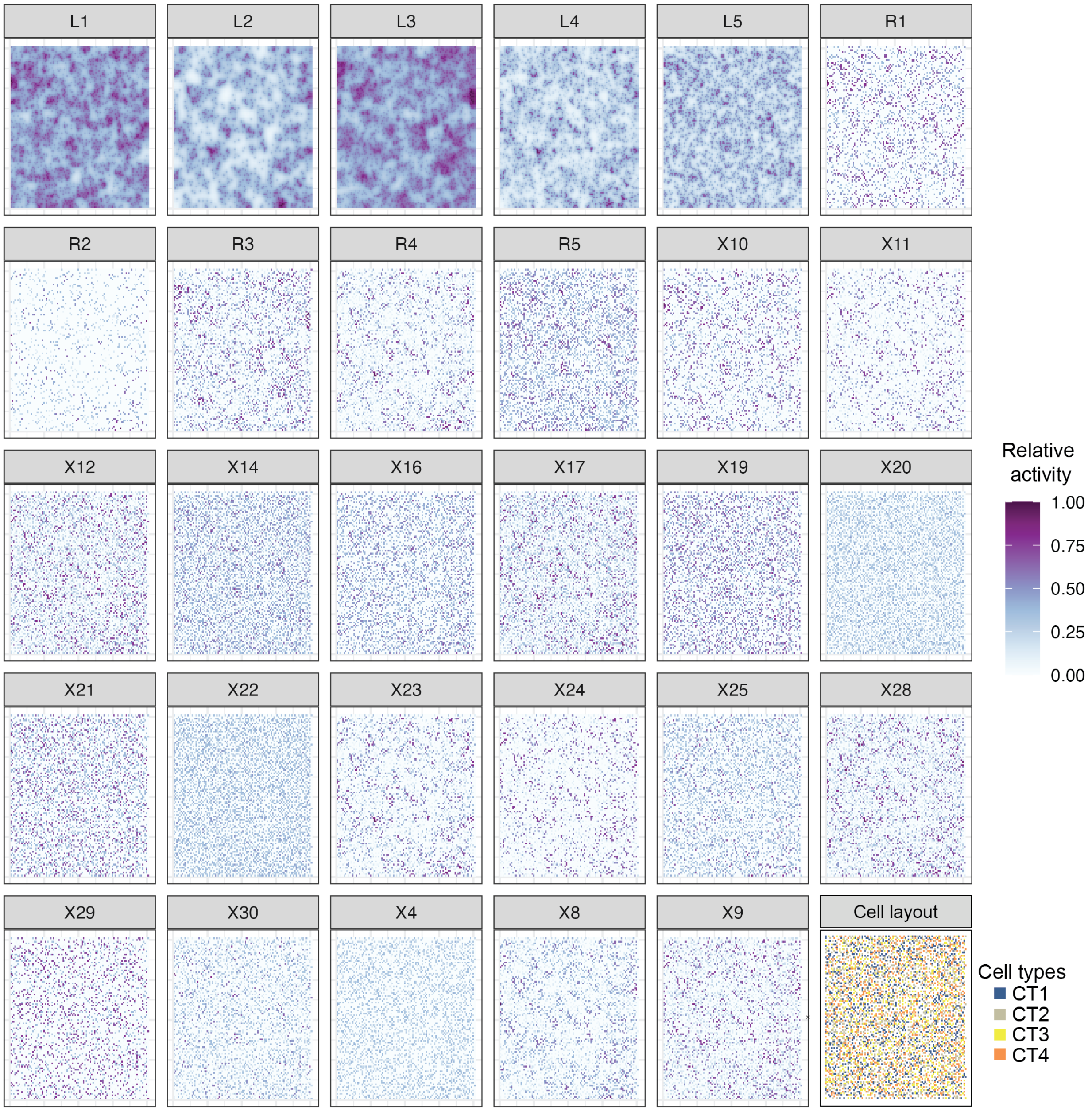

***Fig. S3**. In silico simulation results from a single sample.* *The panels show the distribution of ligands (L1-L5), activity of receptors (R1-R5) and activity of intracellular signaling nodes (X10-X30). The last panel shows the layout of the four cell types.*

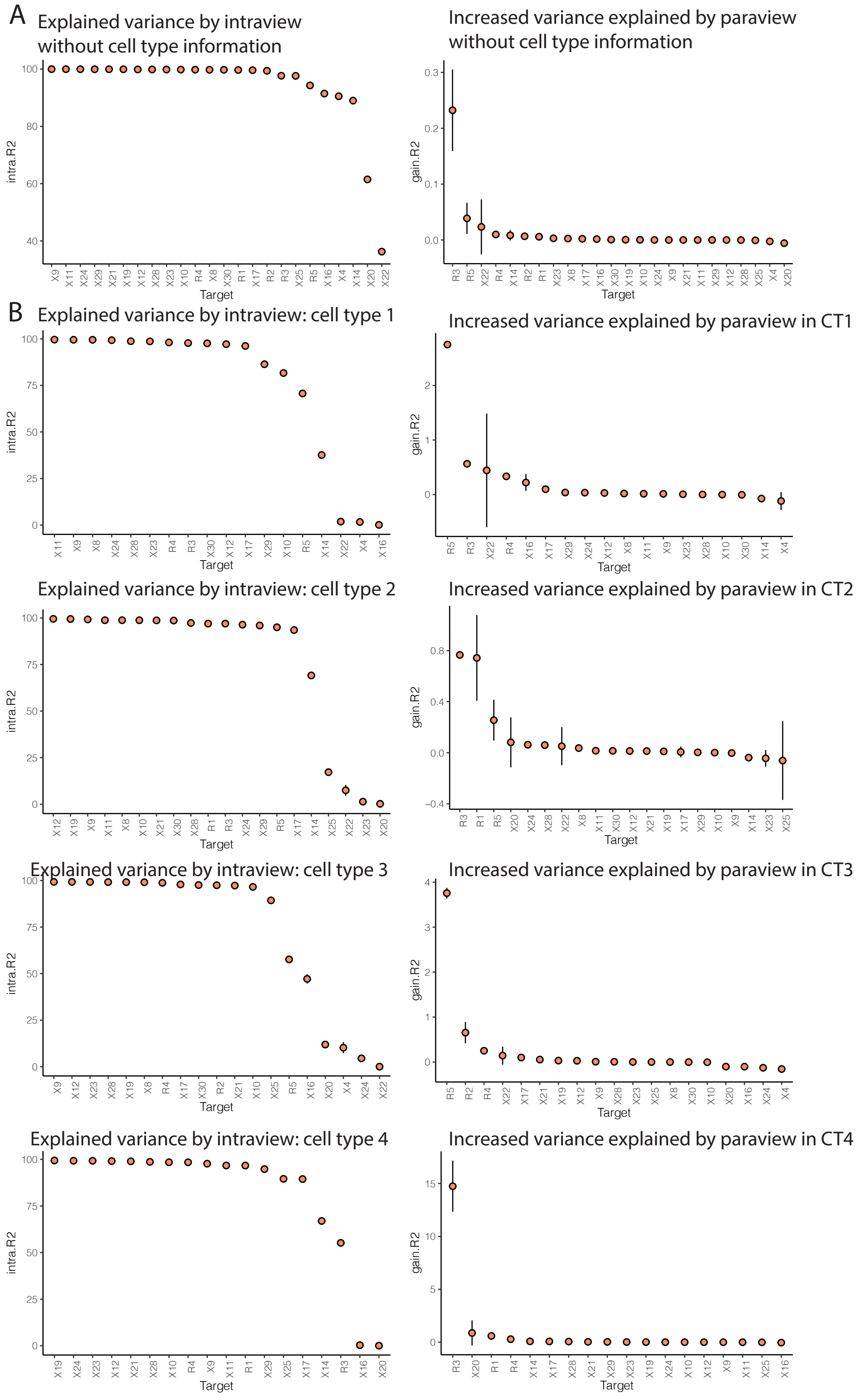

Fig. S4. Explained variance of nodes by the intraview (left) and increased explained variance by the paraview (right) for the scenraio without cell type information (panel A) and with cell type information (panel B).

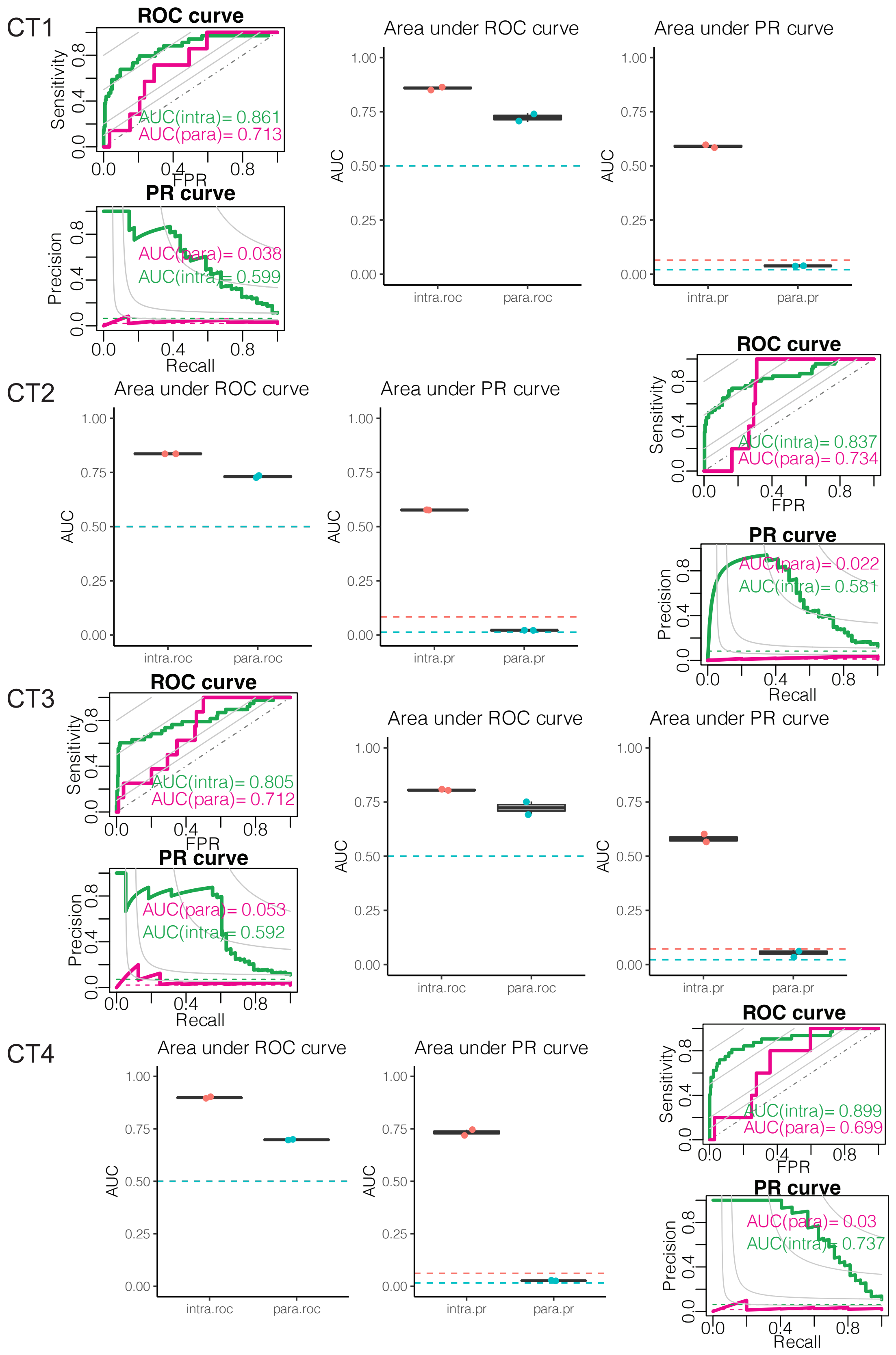

<u>Fig. S5</u>. Distribution of Area Under the Precision-Recall curves (AUPRC) and Receiver-Operating Characteristic curves (AUROC) for the in silico case study, for each cell type (CT1 - CT4).

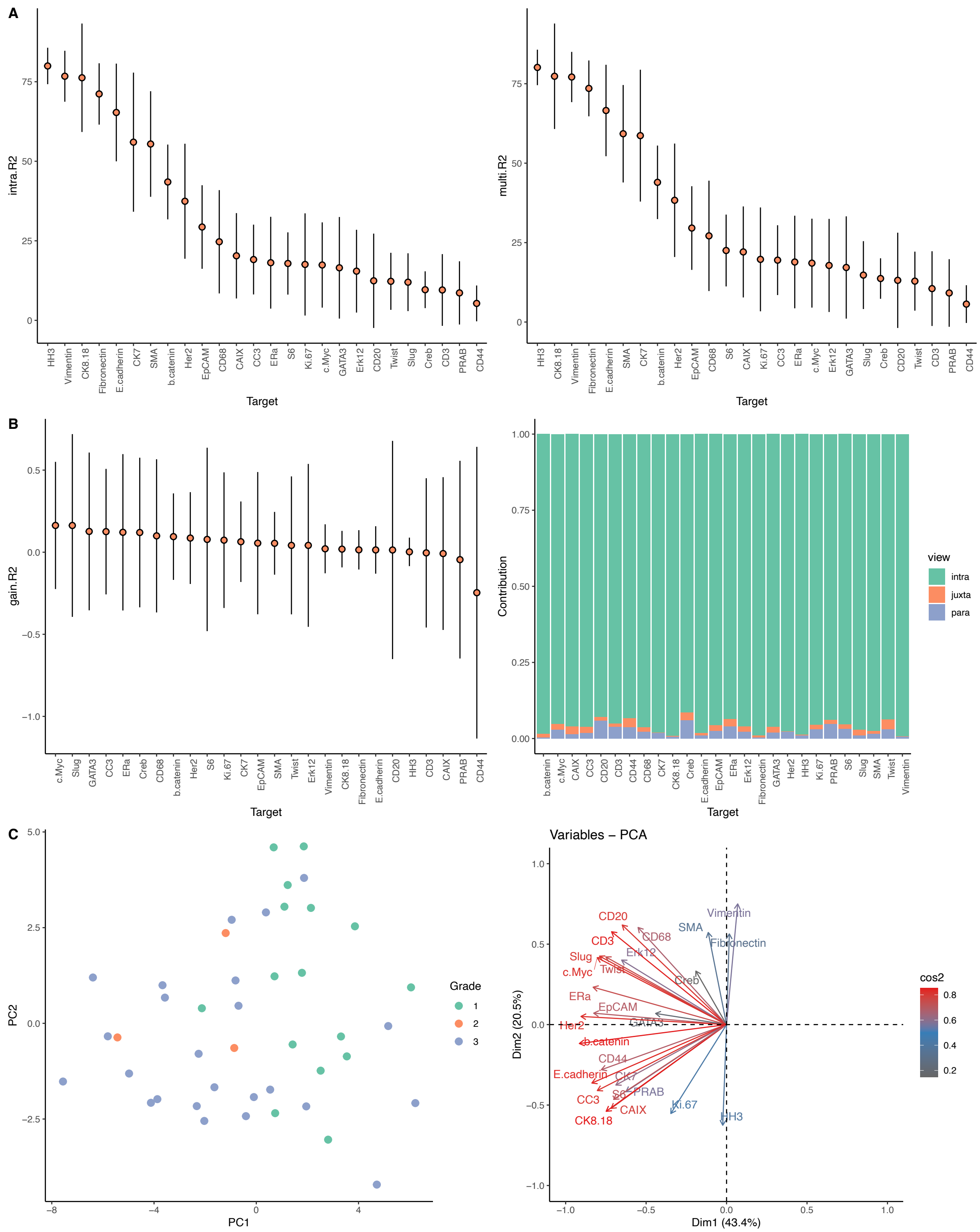

Fig. S6. Additional plots for the IMC data from 46 breast cancer samples. *(A) Predictive performance, in absolute percentage points of variance explained for all samples when considering intraview only (left) and all views together (right). (B) Gain in variance explained and relative contribution of each view to the prediction of the expression of the markers for samples with 10 random permutations of the cell locations. (C) First two principal components of the samples, when represented by the mean expression of the markers across all cells and the importance of the mean expression of the markers in the principal component analysis.*

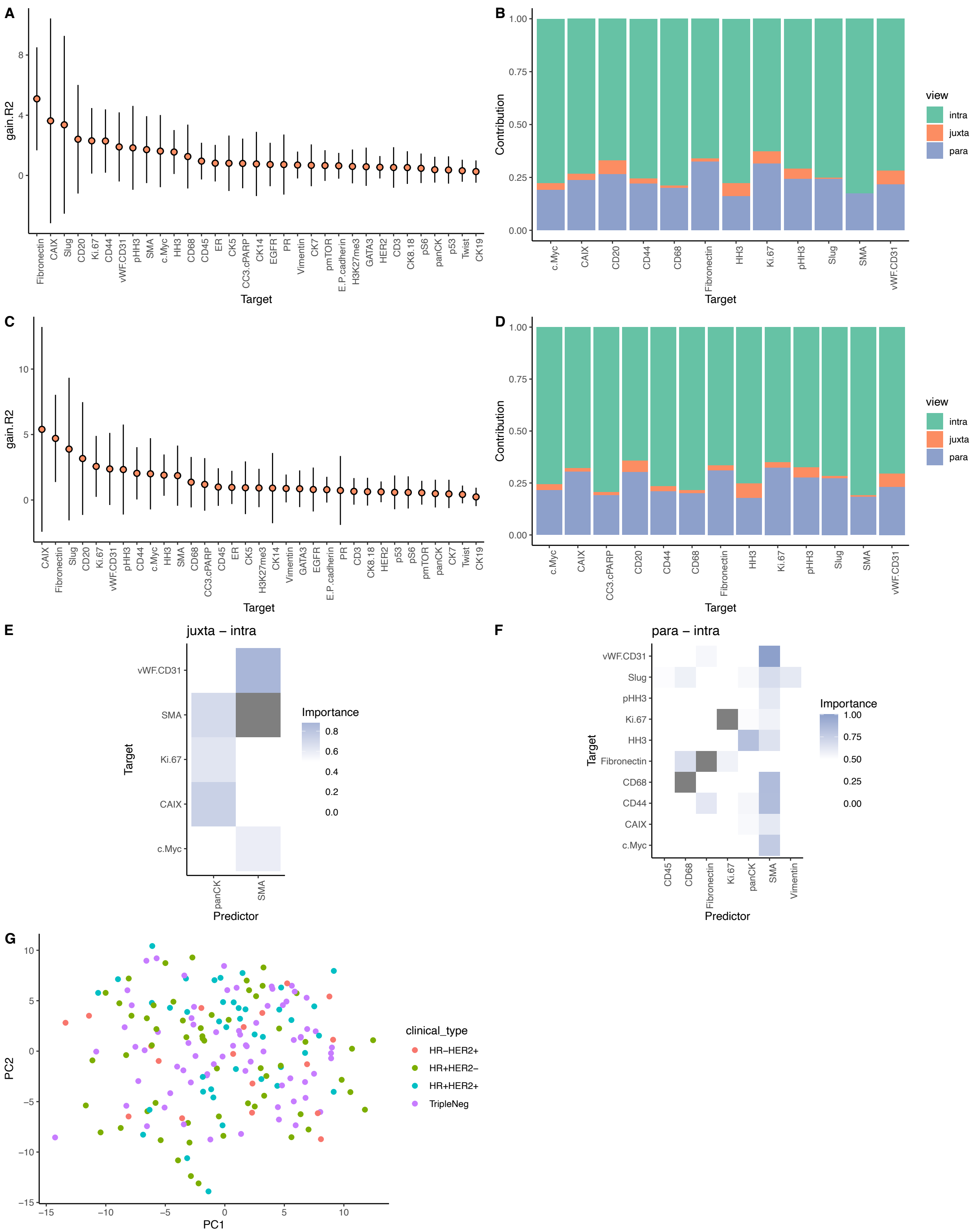
A
gain.R2
Target
Fibronectin
CAIX
Slug
CD20
Ki.67
CD44
vWF.CD31
pHH3
SMA
c.Myc
HH3
CD68
CD45
ER
CK5
CC3.cPARP
CK14
EGFR
PR
Vimentin
CK7
pmTOR
E.P.cadherin
H3K27me3
GATA3
HER2
CD3
CK8.18
pS6
panCK
p53
Twist
CK19
B
Contribution
Target
view
intra
juxta
para
C
gain.R2
Target
D
Contribution
Target
view
intra
juxta
para
E
juxta – intra
Target
Predictor
Importance
vWF.CD31
SMA
Ki.67
CAIX
c.Myc
panCK
F
para – intra
Target
Predictor
Importance
Fibronectin
HH3
CD68
CD45
Vimentin
G
PC1
PC2
clinical_type
HR–HER2+
HR+HER2–
HR+HER2+
TripleNeg

***Fig. S7*. Performance, view contribution and contrasts of IMC data from 415 breast cancer samples.** *(A) Improvement in the predictive performance (variance explained) for all samples when considering multiple views in contrast to a single, intraview (in absolute percentage points). (B) The relative contribution of each view to the prediction of the expression of the markers across all samples. (C) Improvement in the predictive performance (variance explained) for grade 3 samples when considering multiple views in contrast to a single, intraview (in absolute percentage points). (D) The relative contribution of each view to the prediction of the expression of the markers across grade 3 samples. (E) Intragroup contrast of importances of marker expression as predictors of the expression of each target marker between the juxtaview and intraview and (F) paraview and intraview for grade 3 samples. (G) First two principal components of the importance signature of the samples colored by clinical subtype.*

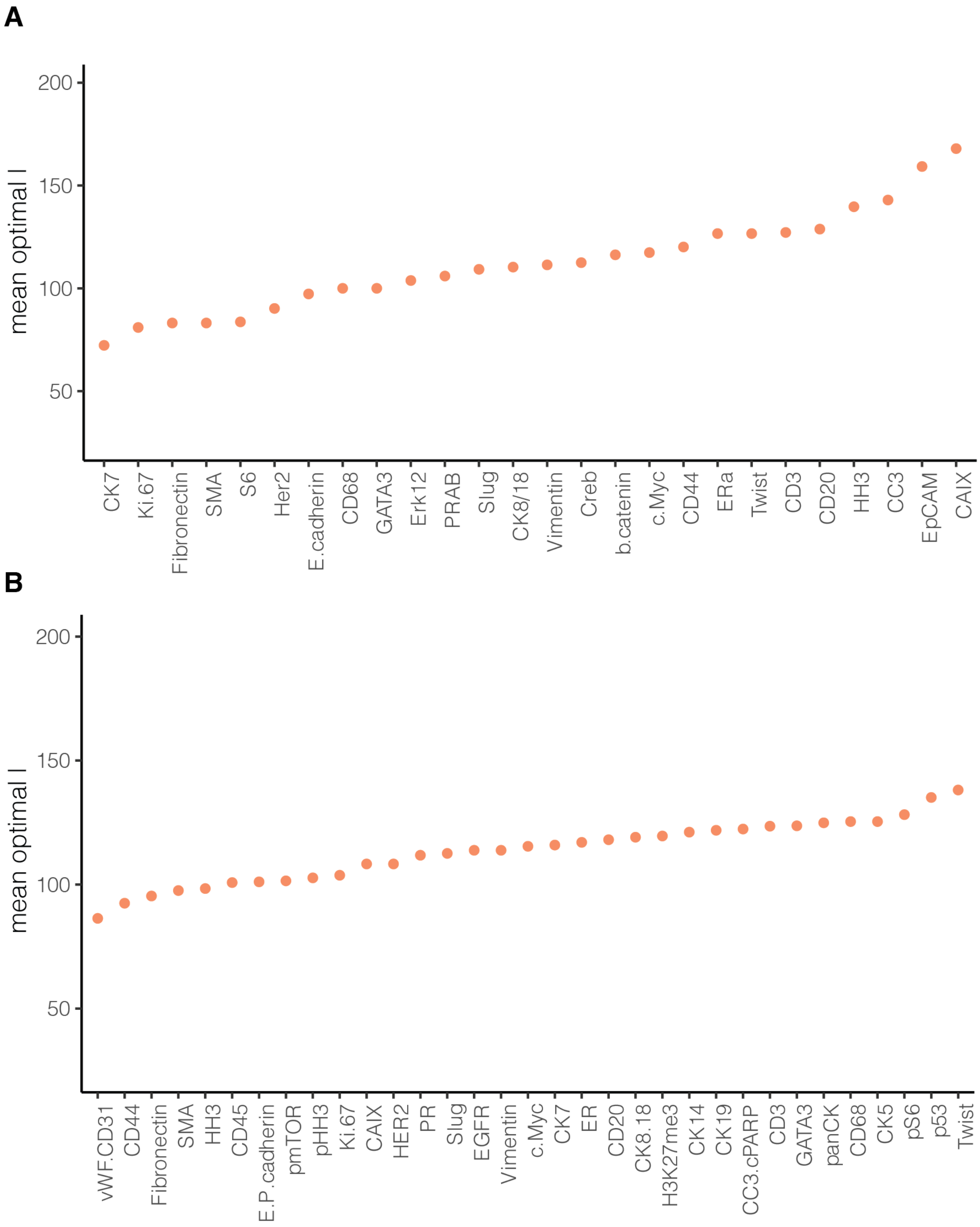

***Fig. S8.* Estimated optimal value for the parameter l** *(A) for the IMC data from 46 breast cancer samples and (B) for the IMC data from 415 breast cancer samples.*

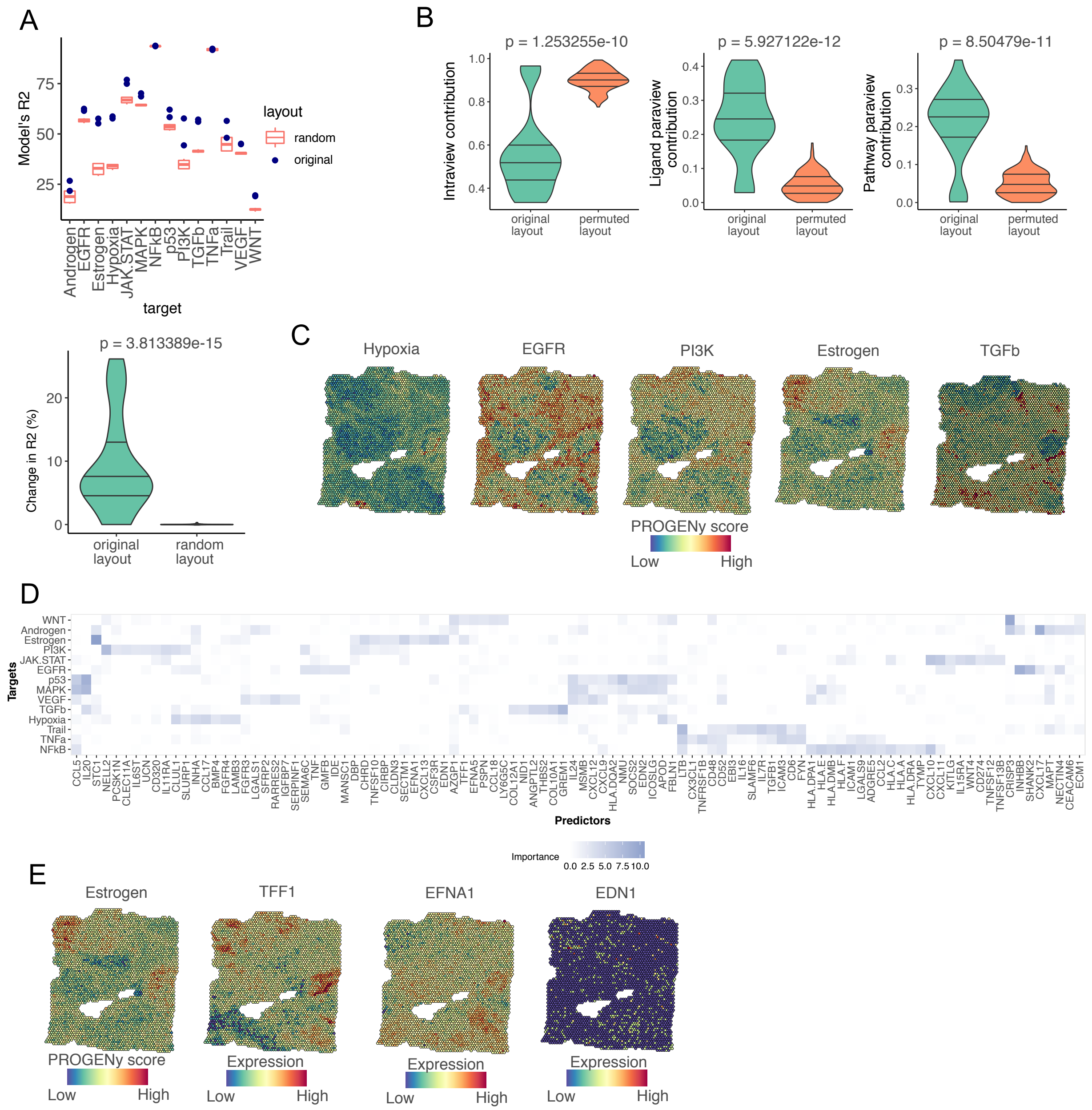

<u>Fig. S9</u>. Application of MISTy to a spatial transcriptomics dataset. *(A) Comparison of MISTy's performance between original slides and a collection of 10 random slides with permuted coordinates. Differences of the R^2 of the multiview model (upper panel) and changes in R^2 between single and multiview models of both classes of models; (B) Differences in view contributions of all predicted markers between models fitted to original or permuted data. (C) Spatial distribution of pathway activities of Hypoxia and its top predictors in the paraview: EGFR, PI3K, Estrogen, and TGFb (D) Variable importances for the ligand paraview. (E) Spatial distribution of Estrogen pathway activities and scaled gene expression of TFF1, EFNA1, and EDN1 (additional top predictors in the ligand paraview).*

Table S1. ***Comparison of methods.*** Methods that can be used to analyze spatially multiplex data, including their requirements.

**Method**	**Model**	**Brief description**		**Technologies**
spatialDE[23], SPARK[21], trendsceek[22]	Various (Gausian processes, Generalized spatial linear models, Marked point processes)	Discovery of spatial patterns of expression of individual markers.		all
histoCAT[31], Giotto[32]	Permutation test	These approaches provide insights into statistically significant structural interactions of pairs of cell types limited to the immediate neighborhood.		all
SVCA[35]	Gaussian Processes	Identification of contributions of different spatial contexts to expression of markers by decomposing the source of variation to three fixed spatial contexts: intrinsic, environmental and intercellular effects.		all
GCNG[33]	Graph Convolutional Neural Networks	Prediction of a relationship between the expression of pairs of genes from graphs of neighborhoods and expression profiles. The model is specific to the task of detection of ligand-receptor interactions. The model requires training with a labeled data of true interactions.		Spatial transcriptomics
MESSI[34]	Mixture of experts	Prediction of a relationship between the expression of a specific set of genes coding for ligands and receptors. The model takes into account the immediate neighborhood and the specific ligand or receptor genes for the specific cell type.		Spatial transcriptomics
**MISTy**	Multi-view learning	Explainable machine learning framework for extracting general structural and functional relationships from any spatial omics data.		all
	**Flexibility of spatial contexts**	**Dependencies between markers**	**Task specific**	**Estimate contributions of spatial contexts**
spatialDE, SPARK, trendsceek	no	no	yes (individual spatial pattern detection)	no
histoCAT, Giotto	no (immediate neighborhood)	yes (paired)	yes (neighborhood composition)	no
SVCA	yes (fixed three)	no	no	yes
GCNG	no (immediate neighborhood)	yes (paired)	yes (ligand-receptor analysis)	no

MESSI	no (immediate neighborhood)	yes (paired)	yes (ligand-receptor analysis)	no
**MISTy**	flexible	yes (multivariate and non linear)	no	yes
	**Minimum input requirements**			
	**Marker expression**	**Spatial coordinates**	**Cell-type annotation**	**Additional**
spatialDE, SPARK, trendsceek	yes	yes	no	no
histoCAT, Giotto	yes	yes	yes	no
SVCA	yes	yes	no	no
GCNG	yes	yes	no	ground truth ligand-receptor interactions
MESSI	yes	yes	yes	cell type specific ligand and receptor coding genes
**MISTy**	yes	yes	no	no
	**Flexibility of analyses**			
	**Structural interactions in the immediate neighborhood**	**Structural interactions in the broader tissue structure**	**Functional interactions in the immediate neighborhood**	**Functional interactions in the broader tissue structure**
spatialDE, SPARK, trendsceek	no	no	no	no
histoCAT, Giotto	yes	no	yes(indirect through cell type annotations)	no
SVCA	yes	yes	no	no
GCNG	no	no	yes (specific)	no
MESSI	no	no	yes (specific)	no
**MISTy**	yes	yes	yes	yes

Table S2. Significant correlation (p <= 0.05) of estimated importances to overall survivability for triple negative samples from the IMC data with 415 breast cancer samples.

**Predictor-Target**	**View**	**Spearman correlation**	**p-value correlation**	**p-value log rank test**
CC3.cPARP-EGFR	intra	0.606	0.003	0.033
Twist-HH3	intra	-0.595	0.004	0.000
Vimentin-HH3	intra	-0.588	0.005	0.023
Vimentin-EGFR	para	-0.587	0.005	0.005
SMA-pHH3	juxta	-0.582	0.005	0.079
Fibronectin-Slug	juxta	0.578	0.006	0.431
CC3.cPARP-Slug	intra	0.577	0.006	0.198
CK8.18-CC3.cPARP	juxta	-0.574	0.006	0.002
pS6-EGFR	intra	0.563	0.007	0.092
CD20-SMA	para	0.563	0.007	0.178
CD44-GATA3	intra	0.554	0.008	0.361
CD45-CD68	juxta	0.548	0.009	0.029
Vimentin-Ki.67	juxta	-0.546	0.010	0.009
H3K27me3-CAIX	para	-0.540	0.010	0.003
CD45-ER	para	0.540	0.010	0.010
Vimentin-CK8.18	juxta	-0.537	0.011	0.014
ER-c.Myc	para	0.532	0.012	0.041
HER2-Fibronectin	juxta	0.531	0.012	0.059
CC3.cPARP-CAIX	intra	0.530	0.012	0.036
CK8.18-Vimentin	para	-0.528	0.013	0.001
SMA-CD20	para	0.527	0.013	0.033
CD20-GATA3	juxta	-0.522	0.014	0.051
pS6-Fibronectin	intra	0.517	0.015	0.230
CD20-GATA3	intra	-0.514	0.015	0.051
HH3-CD45	juxta	0.514	0.015	0.433
CD3-CD45	para	0.506	0.017	0.416
PR-vWF.CD31	intra	0.503	0.018	0.103
CK7-EGFR	intra	-0.496	0.020	0.028

Vimentin-c.Myc	juxta	0.488	0.022	0.008
H3K27me3-Fibronectin	intra	-0.485	0.023	0.175
c.Myc-CK5	para	-0.484	0.024	0.057
CD20-pHH3	para	0.482	0.025	0.055
HER2-Ki.67	intra	0.481	0.025	0.441
CC3.cPARP-GATA3	intra	0.479	0.025	0.005
E.P.cadherin-pHH3	juxta	0.478	0.026	0.003
CD20-Fibronectin	para	0.478	0.026	0.076
CD44-EGFR	intra	-0.471	0.028	0.474
H3K27me3-GATA3	para	0.466	0.030	0.280
H3K27me3-CK8.18	intra	-0.462	0.032	0.008
CAIX-EGFR	intra	0.462	0.032	0.021
c.Myc-GATA3	intra	-0.459	0.033	0.045
ER-CD44	intra	0.459	0.033	0.046
pS6-Ki.67	intra	0.459	0.033	0.076
panCK-SMA	para	-0.459	0.033	0.028
panCK-CK14	juxta	0.457	0.034	0.009
CD45-Slug	intra	-0.456	0.034	0.083
Ki.67-EGFR	intra	-0.455	0.035	0.143
CD44-SMA	para	0.455	0.035	0.216
SMA-HH3	intra	-0.453	0.035	0.000
ER-CK14	juxta	-0.452	0.036	0.002
CAIX-CD20	para	-0.452	0.036	0.319
CK14-GATA3	juxta	0.449	0.037	0.377
vWF.CD31-ER	para	0.447	0.038	0.118
EGFR-Slug	intra	0.444	0.040	0.479
H3K27me3-CAIX	juxta	-0.443	0.040	0.003
panCK-SMA	juxta	-0.441	0.041	0.028
CD44-CD45	para	0.441	0.041	0.009
CD45-CAIX	para	0.440	0.042	0.340
CK19-Vimentin	para	0.440	0.042	0.086

PR-c.Myc	juxta	-0.438	0.043	0.321
CK7-pHH3	juxta	0.434	0.045	0.061
pmTOR-Slug	intra	0.433	0.045	0.016
pS6-HH3	intra	0.430	0.047	0.041
pHH3-CAIX	para	-0.429	0.048	0.002
