# Supplementary material for: Explainable multiview framework for dissecting spatial relationships from highly multiplexed data
Source: Genome Biol. 2022 Apr 14;23:97. doi: 10.1186/s13059-022-02663-5 (PMC9011939; doi:10.1186/s13059-022-02663-5)
Supplement: Supplementary file 2 — Additional file 2. [file 13059_2022_2663_MOESM2_ESM.docx]

Review History

**First round of review**

**Reviewer 1**

**Were you able to assess all statistics in the manuscript, including the appropriateness of statistical tests used?**

No

**Comments to author:**

The present manuscript (GBIO-D-21-01689) describes a computational framework for dissecting spatial interaction between gene/protein markers from multiple views based on IMC data or spatial transcriptional data. The manuscript addressed an interesting topic in an emerging area and would be of broad interest to the community. The novelty of the study lies in modeling and quantification of spatial regulation of gene marker expression. However, the drawback is also obvious, that is, the model lacks enough mechanistic insights.

Major:

1. MISTy does not need cell type annotation, which makes it convenient for analysis but simultaneously suffers from information loss. The title of this paper emphasizes "intercellular signaling", but the cellular source of the extracellular signaling is not reflected by the proposed method, which hampers mechanistic understanding and further analysis.

2. Model formulation (Equation (1)) is a little bit confusing. Could it be better formulated or described? F_0 in Equation (1) was not explained. Furthermore, MISTy was applied to two types of spatial omics data: IMC and 10x ST. These two types of data are different in throughputs and measurement level (protein vs mRNA), which leads to the following issue: the variables of the multi-view models for these two data are conceptually different: one represents marker expression and another represents pathway activity (or ligands as predictors in paraview). So at the first glance it may not be easily accessible to the reader.

3. Regarding multi-views, I have the following questions.
3.1) Does the paraview contain the juxtaview? The manuscript did not clearly state it.
3.2) The intraview and juxtaview (even or paraview) may be correlated, since spatially closer cells may express similar genes/markers/pathways (see: PMID: 31748748). Will this affect the quantification of the contribution of each view to the target expression? This may be reflected in the result of Fig 2E that the increase in explained variance by adding the paraview contribution is small.
3.3) MISTy used linear regression analysis to estimate the contribution of different views. Is this approach too simple? Are there other more reasonable methods to quantify the contribution of different views? For example, could the partial correlation coefficient be used here?

4. The model predictions were difficult to be validated. In the manuscript, the authors only used simulated data at small scale to verify the model. Moreover, the in silico data were simulated for protein measurement, which may not be suitable for mimicking gene expression values in transcriptomic data. In the simulation, the authors considered and distinguished measurements for ligands (ligA, ligB, ligC, and ligD) and ligand producing nodes (prodA, prodB, prodC and prodD), and assumed that "the expression of ligands would be more difficult to capture in a real experiment and therefore excluded them". However, this is not the case for 10x spatial transcriptomic data. Should another in silico data be simulated for the case of the spatial transcriptomic data?

5. In Fig 3E, Fig 4A and Fig 5C-D as well as supp.Fig 4A, the sample in different grade- or subtype- groups were not well separated. The performance of the proposed R2 signature or importance signature is not convincing. Furthermore, what's the superiority of the proposed R2 signature or importance signature? Are they more accurate for classification/prognosis or do they deliver more mechanistic insights? The comparison with other signature is lacking. In addition, in the Abstract section, the authors stated that "the estimated interactions …could improve patient stratification", which, however, was not clearly demonstrated in the Results section.


Minor:
1. For 10x ST data, dimensional reduction was performed by pathway enrichment. In addition to pathway activity, Is it possible to apply MISTy for inter-/ intra-cellular gene-gene relation prediction?

2. P-values should be added to the figures (e.g., supp.Fig 3B, Fig 5E-G, supp.Fig 6A-B).

3. Some text is not concise. For example: P17 L6-16;

4. Some typos have been detected. For instance, Fig 4D legend: "interview" should be "intraview"; Page 22 Line 19-20 "is linked to" should be "are linked to"?

5. In Fig 5E-G, were all selected predictor-target interactions (cutoff 0.5) significantly associated with patient survival? It seems that the figure only shows three pairs of the selected interactions.

6. In the Discussion section, the authors stated that "Our results show that the information that is available from the expression of markers in the broader tissue structure is often more important than their expression in the local cellular niche". But in my view of point, paraview (tissue structure) has broader scope and thus contains more cells and richer signals (although weighted by distance) than the juxtaview (local niche), so the conclusion that the former has more important contribution to the marker expression is trivial.

**Reviewer 2**

**Were you able to assess all statistics in the manuscript, including the appropriateness of statistical tests used?**

Yes

**Comments to author:**

Remarks to the Author:
In this paper, the author developed an explainable multi-view framework, MISTy, for modeling intercellular interactions from any spatial omics data. Unlike existing methods, MISTy takes into account the expression profile from intrinsic effects, local niche, and the broader tissue structure simultaneously, which can deepen our understanding of intercellular communication in a spatial context. However, I have some concerns on the paper:

1. In the paper, there are no performance comparison with existing related methods, which makes the results not very convincing. Although MISTy is not directly comparable to existing methods which only consider local or broader spatial distances, the authors can at least add evaluation on simulated data by intrinsic and local niche view (vs Giotto, histoCAT, MESSI, etc.), the broader tissue view (vs SCVA, GCNG), respectively. Similarly, the author should also compare systematically the estimated interactions with other methods (both spatial-specific methods, such as Giotto, SCVA, GCNG, and traditional methods, such as CellPhoneDB, NicheNet) on a specific dataset, e.g., the triple-negative breast cancer dataset.

In Fig. 5, the authors described the identified interacting structure according to different clinical conditions. These results should be compared with SVCA. Can the interacting relations identified by juxtaview and paraview (such as the juxta CD20-GATA3) be be discovered by SVCA?

2. The literature review is not sufficient. The key contribution of MISTy is using both short and long-range spatial distances for intercellular interaction modeling. However, there are also recent works focusing on this point, e.g., cell2cell [1], Tensor-cell2cell [2], which use Gaussian mixed model to define short-, mid- and long-range distances of cell-cell communication. The authors may also include them for comparison.
[1] Armingol E., Joshi C.J., Baghdassarian H., Shamie I., Ghaddar A., Chan J., Her H.L., O'Rourke E.J., Lewis N.E. Inferring the spatial code of cell-cell interactions and communication across a whole animal body. bioRxiv, (2020). DOI: 10.1101/2020.11.22.392217
[2] Armingol E., Baghdassarian H., Martino C., Perez-Lopez A., Knight R., Lewis N.E. Context-aware deconvolution of cell-cell communication with Tensor-cell2cell bioRxiv, (2021). DOI: 10.1101/2021.09.20.461129

3. SVCA proposed previously by the authors, considers the cell micro-environment, such as local access to oxygen, nutrients etc. Why the environmental effect is not considered in MISTy?

4. Figure 1 is not informative for understanding MISTy in detail. The authors are suggested to add a supplementary figure about the model structure, just like Figure S1 in the SVCA paper. The authors should also add a supplementary table to summarize the difference and strength of MISTy compared with other cell-cell interactions modeling methods.
5. Conventional cell-cell communication tools such as CellPhoneDB and CellChat, need cell clustering (cell-type annotation) as a pre-requisite for subsequent analyses. Understanding which type of cells interact with which type of cells is an important task. Discarding this knowledge is far from true biology. Since the cell-types are common information in the analyses, the authors are suggested to take them into account in the MISTy model as an option.

6. To further validate the performance on prediction of the expression, the authors should provide the results for "Out of Sample Prediction" by cross-validation.

7. The authors first assessed their algorithm in a tissue simulator. Does the simulation process include complex non-linear interactions? In the simulation experiment, the authors used the intrinsic view and paraview to dissecting intercellular signaling. Why does not use the juxtaview here? How to determine which view to use?

8. In Fig. 3E, the authors suggested that the R2 signatures identified by MISTy better captured the variance of the samples compared to the spatial variance signature identified by SVCA. The authors should list these signatures in detail and their names (such as S6_gain.R2) should be explained further.

9. Have the authors considered the randomness of the random forest model? Are the results obtained under multiple runs (or different parameters) consistent with each other?

10. The three views (intraview, juxtaview and paraview) may be correlated to some degrees, the fusion parameters estimated by linear regression may be biased (or unstable, due to multicollinearity). More details on describing how to estimate the fusion parameters and the sensitivities may be added.

Minor
1. Line 35, G_c=∑_(j=1)^n▒〖exp(-(d_ij^2)/j^2 ) (Y_j ) ̃ 〗 , here d_ij might be d_cj.
2. The hyper-parameters (e.g., the number of trees and their depths of the random forest models) adopted by MISTy should be specified in the manuscript.
3. How the fusion parameters (α_v) were normalized?

**Reviewer 3**

**Were you able to assess all statistics in the manuscript, including the appropriateness of statistical tests used?**

Yes

**Comments to author:**

In this work, Tanevski and colleagues propose a method called MISTy which uses spatial transcriptomics data to dissect for each gene or module of genes the contributions of its variation from intra-cellular, local neighborhood and larger neighborhood. For each view MISTy measures the variability captured and the relative contribution of each view for each marker can then be examined. They demonstrate the method using a simulation. They then apply it to a breast cancer dataset in which interactions are actually associated with clinical parameters. An advantage of this method is that it requires no assumptions about that data - for example there is no need for cell type annotation. The method allows for exploration which will be very useful for the community. I think that this method seems to be extremely useful. I am very motivated to test it out.

I do have the following suggestions for the authors to consider:


1. Readability. This will be a difficult read for biologists. Figure 1 is an opportunity to provide an intuition for the method, but it is not well used. The notion of dissecting the sources of variation across the views is a core concept of the paper yet this is not explained by the paper, nor is it well explained in the text. I recommend adding a schematic with examples. Also the notion of feature importance is not well explained in the text. There should be a schematic to also describe this.

2. The simulation is an interesting demonstration of MISTy however the results are discussed only in the text. It would be useful to also establish the expected results for each of the genes shown in Figure 2B and then compare these to the results of the simulation. The authors can establish an expectation that ECM for example would only be detected as capturing variance in the paraview. They can rank the genes and compare with the observed results in the simulation.

3. It is very interesting that the authors can compare samples according to the variance captured across the three views of the 26 markers (Figure 3 analysis). The authors claim that there is structure in the PCA but they do not quantify this. Also they describe a set of detected markers that contribute most to the PCs - pS6, CAIX, Erk12, and Cytokeratin 7 - but they do not describe what has been previously revealed about these.

4. The authors should also provide a test for whether it is subtypes (rather than grade) of breast cancer that is being captured in the analysis shown in Figure 3E. They could just color by subtypes in the same analysis shown there. Structure according to subtypes would also be interesting.

5. In the Figure 5 analysis shown in E-G, only three gene pairs are shown. Are these cherry-picked from the full set of interactions? Would other pairs also show a significant change or at least a change in the same direction for survivability?

6. In Figure 6, the authors detect a relationship between pathways; for example the TNFa and NFkB pathways. Did the authors control for the fact that these pathways may have identical genes? A lack of independence across the gene sets would of course skew the results.

7. Figure 6 shows the primary data for Visium but the same is not shown for the breast cancer datasets. Including this in FIgure 3 or 4 could greatly improve the readability of the paper.

8. Minor comments: In Figure 2B there is an orange circle that is out of place?

**Authors Response**

**Point-by-point responses to the reviewers’ comments:**

The present manuscript (GBIO-D-21-01689) describes a computational framework for dissecting spatial interaction between gene/protein markers from multiple views based on IMC data or spatial transcriptional data. The manuscript addressed an interesting topic in an emerging area and would be of broad interest to the community. The novelty of the study lies in modeling and quantification of spatial regulation of gene marker expression. However, the drawback is also obvious, that is, the model lacks enough mechanistic insights.

*We are glad to read that the reviewer considers our manuscript of broad interest to the community and that it addresses an interesting topic, providing novel modeling strategies. With regard to the mechanistic (functional) insight, we developed MISTy as a flexible approach adaptable to specific biological questions from which we believe mechanistic insight can be gained. For example, in Figure 7 (previously Figure 6) we elaborated on the relationships between signaling pathway activities and ligand’s expression in spatial transcriptomics data. We aimed to show that the use of MISTy isn’t limited to the relations between a collection of “unrelated” markers and that MISTy can be coupled to prior knowledge resources and functional transcriptomic tools to generate hypotheses of tissue function and organization. Additionally, in our extended in silico study we now demonstrate the degree of structural and mechanistic insights that can be gained. We also thank the reviewer for her/his comments that we address below:*

Major: 1. MISTy does not need cell type annotation, which makes it convenient for analysis but simultaneously suffers from information loss.

*We agree that information on cell type annotation, in the cases where this is available and of good quality, is valuable and should not be neglected. While MISTy can leverage cell type information, it does not necessarily need it. This we consider an important strength of MISTy. To demonstrate MISTy’s capability to use cell type as well as marker information, we extended the in silico study to demonstrate how MISTy can highlight both structural and functional relationships with and without using both types of information. This highlights the flexibility of MISTy and provides insights into advantages and disadvantages of using cell type vs. marker information.*

The title of this paper emphasizes "intercellular signaling", but the cellular source of the extracellular signaling is not reflected by the proposed method, which hampers mechanistic understanding and further analysis.

*Indeed, in hindsight the emphasis in intercellular signaling in the title was misleading. MISTy is a general-purpose framework to study interactions among spatial units (cell or spot) in spatially-resolved data. These can be related or not to intercellular signaling. We changed the title to spatial relationships. We also further elaborate in the text (end of Results subsection “MISTy: Multiview intercellular spatial modeling framework”) on the relationships that MISTy is capturing. In the revised version we give examples of more and different use cases, the type of information captured and the interpretation of the results of each use case.*

2. Model formulation (Equation (1)) is a little bit confusing. Could it be better formulated or described? F_0 in Equation (1) was not explained.

*We updated the description of the model, we also included examples of pipelines and a new figure (Supplementary Figure 1) to better describe the view generation and modeling process.*

Furthermore, MISTy was applied to two types of spatial omics data: IMC and 10x ST. These two types of data are different in throughputs and measurement level (protein vs mRNA), which leads to the following issue: the variables of the multi-view models for these two data are conceptually different: one represents marker expression and another represents pathway activity (or ligands as predictors in paraview). So at the first glance it may not be easily accessible to the reader.

*As the reviewer correctly notes, MISTy’s model and analyses quite different molecular/biological meaning in the case studies, since these are based on different technologies. We specifically chose case studies in these two main areas of spatial omics technologies - spatial (antibody-based) proteomics and spatial transcriptomics - to demonstrate MISTy’s flexibility. Indeed, the corresponding results represent different insights, so we now clarified the differences in the text in each results subsection.*

*We further address this in the revised version by extending the existing information in the Results section and later explaining better the specific issue of importances and aggregation in more detail in the Methods section, in particular in the “importance weighting and aggregation” subsection.*

3. Regarding multi-views, I have the following questions. 3.1) Does the paraview contain the juxtaview? The manuscript did not clearly state it.

*The reviewer is correct: in the original manuscript it was not clear that the juxtaview and the paraview shared, in part, the same expressions. More specifically, the paraview contained the expressions from the immediate neighborhood with a relatively high weight and complemented it with the expression coming from the broader tissue structure with decaying weight values. The combination of the predictions with the meta-model in principle corrected for this, and contrasting of the results could be used to highlight the interactions that are specific to the juxtaview or the paraview.*

*This can be a limitation and thus in the revised version of the manuscript and in the new version of MISTy’s implementation we integrated a more general solution. In particular, we allow for explicit exclusion of a region (zone of indifference) from the paraview. In this way we ensure that the results are not ambiguous. All experiments that included juxtaview and paraview (IMC) were rerun with the updated version where the zone of indifference for the paraview was defined to be equal to the region of space captured by the juxtaview. We also noted this change in the Results section and in the Methods section in the subsection “View generation”.*

*Of note, the observed change in results was not major. Qualitatively we didn’t observe deviation from the conclusions from the previous version of the manuscript. Quantitatively, in the view contribution results we observed an increase in the contribution of the paraview and decrease in the contribution of the juxtaview (Figure 4), strengthening our conclusion of the importance of considering the broader tissue structure in addition to the immediate neighborhood.*

3.2) The intraview and juxtaview (even or paraview) may be correlated, since spatially closer cells may express similar genes/markers/pathways (see: PMID: 31748748). Will this affect the quantification of the contribution of each view to the target expression? This may be reflected in the result of Fig 2E that the increase in explained variance by adding the paraview contribution is small.

*We agree and we make sure to capture and reflect this in the results of MISTy. Since the intraview contains more information than the juxtaview, even in the case of correlation due to spatial proximity of similar cells, the meta-model will prefer the contribution of the intraview over the contribution of the juxtaview. Since the model is regularized (see response to the next point) this preference will be even more significant. Furthermore, if the immediate neighborhood contains cells with similar expressions and this is captured by the juxtaview and therefore improving the predictive performance of the model, then this correlation is meaningful and should be taken into account when interpreting the results. Following, if present, this effect will also be noticeable in the similarity of the estimated predictor-target importance in the intraview and juxtaview. In other words, if the cells in the juxtaview express the same genes in the same way, this information is relevant and MISTy will capture it. Contrasting juxtaview with intraview will remove this and will highlight only interactions that come from the juxtaview. In the results presented in the manuscript this was not the case, and to illustrate the point, now in Supplementary Figure 7 we show the contrast of estimated interactions between the juxtaview and the intraview for the large IMC dataset.*

3.3) MISTy used linear regression analysis to estimate the contribution of different views. Is this approach too simple? Are there other more reasonable methods to quantify the contribution of different views? For example, could the partial correlation coefficient be used here?

*We agree that in case of highly correlated views, this might be an issue. Therefore to address this, in the new implementation we are regularizing the meta-model. While partial correlation coefficient might address this issue as the reviewer suggests, an approach that is better suited to our setting is ridge regression. Ridge regression is a well established approach to addressing issues of multicolinear or correlated predictors in multiple linear regression. We note this change in the Results section of the revised manuscript and we rerun all experiments using ridge regression to train the meta-model.*

4. The model predictions were difficult to be validated. In the manuscript, the authors only used simulated data at small scale to verify the model. Moreover, the in silico data were simulated for protein measurement, which may not be suitable for mimicking gene expression values in transcriptomic data. In the simulation, the authors considered and distinguished measurements for ligands (ligA, ligB, ligC, and ligD) and ligand producing nodes (prodA, prodB, prodC and prodD), and assumed that "the expression of ligands would be more difficult to capture in a real experiment and therefore excluded them". However, this is not the case for 10x spatial transcriptomic data. Should another in silico data be simulated for the case of the spatial transcriptomic data?

*We agree that the model used was of small scale, and indeed it described proteins, as these are the major players of signaling; this way we can directly look at cell-cell communication and have a ground truth. The correlation between proteins and transcripts is limited and largely unknown (Liu, Beyer & Aebersold Cell 2016), and therefore such an in silico model could not be directly used for spatial transcriptomics. For this reason, we now added a second simulated case study for spatial transcriptomics, as the reviewer suggested (see Results and new Figure 2). We show the performance of MISTy on the tasks of recovering the structural relationships and functional relationships separately, in the new and previous in silico models, respectively. Furthermore, we increased the scale of the existing in silico models and provide more detailed results.*

5. In Fig 3E, Fig 4A and Fig 5C-D as well as supp.Fig 4A, the sample in different grade- or subtype- groups were not well separated. The performance of the proposed R2 signature or importance signature is not convincing. Furthermore, what's the superiority of the proposed R2 signature or importance signature? Are they more accurate for classification/prognosis or do they deliver more mechanistic insights? The comparison with other signature is lacking.

*We agree that this was not clear. In the Results section we now explain in more detail what constitutes a MISTy results signature. Note that we also use the R2 signature to be able to compare to the same type of signature available from SVCA results and extend the comparative analysis. In addition, since MISTy offers a richer set of outputs, we are also able to extract view contributions and importance signatures. These signatures and the structure of the results allows us to better focus our exploratory and comparative analysis of the samples.*

*Furthermore, we now state in the manuscript the results compared to the mean expression, where we see a less informative reduction of expression during tumor progression uniformly across all markers. In the R2 and importance signatures we identify clearer and more informative relationship between the availability of information coming from the different spatial contexts and tumor progression.*

In addition, in the Abstract section, the authors stated that "the estimated interactions …could improve patient stratification", which, however, was not clearly demonstrated in the Results section.

*We agree that this wording was not accurate and we modified the sentence in the abstract to “We estimated structural and functional interactions coming from different spatial contexts in breast cancer and demonstrated how to relate MISTy’s results to clinical features.” Having said that, we do demonstrate in this manuscript that the presence / absence of specific interactions is significantly linked to patient outcome, which could be used for patient stratification in a prognostic study setup.*

Minor: 1. For 10x ST data, dimensional reduction was performed by pathway enrichment. In addition to pathway activity, Is it possible to apply MISTy for inter-/ intra-cellular gene-gene relation prediction?

*Yes, this is indeed possible. An example of this type of workflow is shown in the extended in-silico experiment where we use the expression of the simulated 100 genes to estimate inter cellular relationships between them. As the amount of interactions to be interpreted is large even in this case, we had to summarize the findings by relating the estimated importances to the cell-type markers in Figure 2E. In a workflow where thousands of genes are measured the visual analysis of a large matrix can become tedious. In addition some false positive interactions might skew the downstream analysis. That is why in this case we used, dimensionality reduction by functional footprint estimation (pathway or transcription factor activities), which provides a compressed representation that allows for more focused functional interpretation. We have previously shown that these methods can be applied to scRNA-seq data despite not having whole-genome coverage (Holland et al., Genome biology, 2020).*

2. P-values should be added to the figures (e.g., supp.Fig 3B, Fig 5E-G, supp.Fig 6A-B).

*We added the p-values in the figures.*

3. Some text is not concise. For example: P17 L6-16;

*We revised the text and tried to improve the readability.*

4. Some typos have been detected. For instance, Fig 4D legend: "interview" should be "intraview"; Page 22 Line 19-20 "is linked to" should be "are linked to"?

*We fixed these typos.*

5. In Fig 5E-G, were all selected predictor-target interactions (cutoff 0.5) significantly associated with patient survival? It seems that the figure only shows three pairs of the selected interactions.

*We revised the text to read “We grouped the samples by the estimated importance of the selected predictor-target interaction. If the estimated importance for that predictor-target interaction in that sample is larger than 0.5 we consider that sample to be in the positive group, otherwise we consider the sample to be in the negative group.” Not all estimated importances of the predictor-target pairs are associated with patient survival. We considered the predictor target pairs with significantly correlated importances to overall survival in triple-negative grade 3 tumor samples. In the figure we showed the KM curves only for the most correlated predictor-target pair per view. In the revised manuscript this is shown in Supplementary Table 2 where we also added information about the p-value of the log rank test for the KM curves for all pairs.*

6. In the Discussion section, the authors stated that "Our results show that the information that is available from the expression of markers in the broader tissue structure is often more important than their expression in the local cellular niche". But in my view of point, paraview (tissue structure) has broader scope and thus contains more cells and richer signals (although weighted by distance) than the juxtaview (local niche), so the conclusion that the former has more important contribution to the marker expression is trivial.

*Indeed, this is an intuitive result, although we note that no other method quantifies nor considers this. We make this point now in the discussion, by adding the following sentence: Of note, this result, which is biologically intuitive, could not be found with previous methods that do not distinguish between para- and juxtaview.*

Reviewer #2:

Remarks to the Author: In this paper, the author developed an explainable multi-view framework, MISTy, for modeling intercellular interactions from any spatial omics data. Unlike existing methods, MISTy takes into account the expression profile from intrinsic effects, local niche, and the broader tissue structure simultaneously, which can deepen our understanding of intercellular communication in a spatial context.

*We thank the reviewer for underscoring the novelty of MISTy, and for their suggestions and comments, that we hope to have addressed as outlined below.*

However, I have some concerns on the paper:

1. In the paper, there are no performance comparison with existing related methods, which makes the results not very convincing. Although MISTy is not directly comparable to existing methods which only consider local or broader spatial distances, the authors can at least add evaluation on simulated data by intrinsic and local niche view (vs Giotto, histoCAT, MESSI, etc.), the broader tissue view (vs SCVA, GCNG), respectively. Similarly, the author should also compare systematically the estimated interactions with other methods (both spatial-specific methods, such as Giotto, SCVA, GCNG, and traditional methods, such as CellPhoneDB, NicheNet) on a specific dataset, e.g., the triple-negative breast cancer dataset.

*In the manuscript, even in the title, we had positioned MISTy as a tool for cell-cell communication which we acknowledge was misleading; now we refer to MISTy as a tool for spatial relationships, and we changed the title accordingly. By placing MISTy in the communication realm, it was a fair and natural question from the reviewer to ask for comparison with tools that analyze communication. We elaborate below the differences, as well as in a new Supplementary Table 1.*

*There are indeed a large number of valuable methods to study cell-cell interactions and more specifically cell-cell communication. The two methods that are directly comparable to MISTy are histoCAT and SVCA were run on the same data for comparison. histoCAT provides the initial analysis, which was re-analyzed by SVCA afterwards. We added our own results and compared them to the published findings - highlighting that we recapitulate published results as well as extend them with MISTy.*

*Other methods like MESSI and GCNG are focused on ligand-receptor interactions. GCGN requires a "ground truth" labeled data for supervised training, and using this find ligand-receptor paris. MISTy in contrast doesn’t require training or other types of bias and does not look specifically at ligand-receptor pairs. Similarly, MESSI requires at input known ligand-receptor pairs to learn relationships at the intra and intercellular level, and requires known cell types. A further noteworthy difference is that both GCGN and MESSI are specific to spatial transcriptomics while MISTy is a general-purpose framework, as we shown in the manuscript running it on IMC besides spatial transcriptomic data.*

*Giotto allows to estimate co-occurrence of cell-types by performing an enrichment analysis over a spatial neighborhood. Similarly to histoCAT, this requires cell-type labeling, which MISTy doesn’t. Additionally, Giotto allows to estimate the spatial co-expression of pairs of markers in neighboring cells from two different classes. MISTy, in comparison, isn’t limited to linear relationships between pairs of markers, but rather allows to relate a single marker to a collection of predictors. Moreover, while MISTy can work without cell-type labels, these can be used to create specific cell-type predictor views, which highlights the benefits of our flexible framework. cell2cell allows for the estimation of communication events between pairs of cells using modified Bray-Curtis scores, under the assumption that cells that are closer to each other have a greater complementation of ligand receptor pairs. cell2cell coupled to a genetic algorithm can identify the most informative ligand-receptor pairs in regards to the observed anti-correlation between the defined cell-cell interaction scores and euclidean distance. MISTy, in contrast, is not focused in scoring cell to cell (or location to location) interactions, but in marker dependencies in different spatial contexts. In that regard, we encode the importance of proximity in marker dependencies in the estimation of the different spatially contextualized views. As a possible use case pipeline, one could use the interaction scores proposed by cell2cell to penalize the importance of locations in the estimation of the spatially contextualized views.*

*Tensor-cell2cell is an approach to contrast communication events between different cellular contexts (time-points, tissue areas, etc.) from single cell data using Tensor Component Analysis (TCA). Even though we showed how MISTy’s outputs can be used to classify breast cancer samples from IMC data, our model doesn’t explicitly take into account multiple conditions. Rather, we create signatures per slide and use those with classical dimensionality reduction approaches such as PCA or statistical tests for population comparisons. A complementary approach to use Tensor-cell2cell with spatial data would be to use MISTy’s importances signatures as the input communication score matrices for Tensor-cell2cell.*

*That being said, we think that the novel ideas presented in tools such as MESSI, GCCG, cell2cell and tensor-cell2cell are complementary to our work and should be highlighted, and we now accordingly do so in the Discussion.*

*Finally, there are a number of methods such as CellPhoneDB, NicheNet to study cell-cell communication on specific samples without modeling the spatial location. These methods are complementary to MISTy, and one could for example run CellPhoneDB and NicheNet on the spots from the 10x Visium data, and then run on the resulting features MISTy among the spots. We recently developed LIANA (https://saezlab.github.io/liana/) as a framework to integrate different ligand-receptor methods that could be then used together with MISTy to perform such analyses.*

*In summary, MISTy is a framework for unbiased exploratory estimation of interactions and hypothesis generation, that is applicable in general to any spatially resolved data and doesn't rely on any additional source of information at input, complementing other existing methods. MISTy’s flexibility allows it to build complex pipelines that can incorporate specific properties of the spatial technology in hand that other tools ignore, e.g. the study of the dependencies between signaling pathway activities and ligand expression in spatial transcriptomics as shown in Fig. 6.*

In Fig. 5, the authors described the identified interacting structure according to different clinical conditions. These results should be compared with SVCA. Can the interacting relations identified by juxtaview and paraview (such as the juxta CD20-GATA3) be discovered by SVCA?

*While SVCA indeed is the closest method to MISTy, the comparison is not possible because (i) such a large case study can not be run as the Guassian-process-based implementation of SVCA does not scale up (running SVCA on a single slide of the in silico data takes more than 24 hours compared to tens of seconds with MISTy), and (ii) SVCA does not define interactions, only contributions, so that we could not compare those results.*

2. The literature review is not sufficient. The key contribution of MISTy is using both short and long-range spatial distances for intercellular interaction modeling. However, there are also recent works focusing on this point, e.g., cell2cell [1], Tensor-cell2cell [2], which use Gaussian mixed model to define short-, mid- and long-range distances of cell-cell communication. The authors may also include them for comparison. [1] Armingol E., Joshi C.J., Baghdassarian H., Shamie I., Ghaddar A., Chan J., Her H.L., O'Rourke E.J., Lewis N.E. Inferring the spatial code of cell-cell interactions and communication across a whole animal body. bioRxiv, (2020). DOI: 10.1101/2020.11.22.392217 [2] Armingol E., Baghdassarian H., Martino C., Perez-Lopez A., Knight R., Lewis N.E. Context-aware deconvolution of cell-cell communication with Tensor-cell2cell bioRxiv, (2021). DOI: 10.1101/2021.09.20.461129

*We thank the reviewer for pointing at these methods. We included these methods along with the other in our response to the previous point 1 of the reviewer and cited them in the Discussion section.*

3. SVCA proposed previously by the authors, considers the cell micro-environment, such as local access to oxygen, nutrients etc. Why the environmental effect is not considered in MISTy?

*The environmental effect is captured implicitly by the MISTy model in the intercept term of the meta model for each view and each target marker. We now clarified this in the Results section. Additionally, the paraview constructed with a gaussian family of weights, although weighing significantly only a limited radius of expression it also captures information from the whole slide.*

4. Figure 1 is not informative for understanding MISTy in detail. The authors are suggested to add a supplementary figure about the model structure, just like Figure S1 in the SVCA paper.

*We thank the reviewer for this valuable suggestion and we now include a more detailed visual overview of MISTy in Supplementary Figure 1.*

The authors should also add a supplementary table to summarize the difference and strength of MISTy compared with other cell-cell interactions modeling methods.

*We agree that such a table can be useful, and in the revised manuscript we include it as Supplementary Table 1.*

5. Conventional cell-cell communication tools such as CellPhoneDB and CellChat, need cell clustering (cell-type annotation) as a pre-requisite for subsequent analyses. Understanding which type of cells interact with which type of cells is an important task. Discarding this knowledge is far from true biology. Since the cell-types are common information in the analyses, the authors are suggested to take them into account in the MISTy model as an option.

*Indeed cell-type information if available is helpful and could be used, as also commented by reviewer 1 as a first comment. While MISTy does not need cell type information, it can use it.*

*To demonstrate MISTy’s capability to use cell type as well as marker information, we extended the in silico study to demonstrate how MISTy can highlight both structural and functional relationships with and without using both types of information. For this we added results of cell type specific workflows in the mechanistic in silico experiments in order to show the influence of the availability of such information on the performance of MISTy workflows. The IMC experiments due to the use of cell type markers are related to the recovery of cell type based structural relationships.*

6. To further validate the performance on prediction of the expression, the authors should provide the results for "Out of Sample Prediction" by cross-validation.

*All the results reported in the manuscript relating to the performance of view and target specific models are obtained by “out-of-bag” predictions, which are pessimistic estimates of the expected generalization performance of the model. Out of sample performance estimation is one of the requirements for the choice of an algorithm highlighted in the Results section. Additionally, the performance of the meta-model (multi-view and gain od variance explained) that is reported in the results is the mean performance from 10-fold cross validation. We now added this missing information in the Results section.*

7. The authors first assessed their algorithm in a tissue simulator. Does the simulation process include complex non-linear interactions?

*The tissue simulator is based on a discretized (in space and time) reaction diffusion system as summarized in Methods (In silico mechanistic model, Eq2). At each spot, the activation of a species is described by linear equations, however, the whole dynamic model is nonlinear due to the diffusion terms.*

In the simulation experiment, the authors used the intrinsic view and paraview to dissecting intercellular signaling. Why does not use the juxtaview here? How to determine which view to use?

*Since the mechanistic in silico model does not include signaling by direct physical interaction between cells, we decided to not include juxtaview in the pipeline. This also simplified the analysis and the demonstration of the modeling framework. In general, the Misty framework allows the user to decide about the types of views to include in the analysis. This allows the user to tailor the model to the knowledge about the system and the question of interest, as the in silico case illustrates.*

8. In Fig. 3E, the authors suggested that the R2 signatures identified by MISTy better captured the variance of the samples compared to the spatial variance signature identified by SVCA. The authors should list these signatures in detail and their names (such as S6_gain.R2) should be explained further.

*In the revised manuscript we described the naming of the variables in the signature plots in more detail. We also explained in more detail what constitutes a MISTy result signature in the text of the Results section.*

9. Have the authors considered the randomness of the random forest model? Are the results obtained under multiple runs (or different parameters) consistent with each other?

*Indeed a random forest can give different results due to randomness. To address this, ensembling is widely known as one of the best approaches; the higher the number of bootstraps the more stable the prediction. Adding the component of random selection of variables at each split reduces the error due to variance and increases the stability. In the referenced paper (Brieman, Machine Learning, 2001) there is an empirical and theoretical analysis of the behavior of the model including stability, which guarantees that the chosen parameters provide consistent results. This is explained now in the manuscript.*

10. The three views (intraview, juxtaview and paraview) may be correlated to some degrees, the fusion parameters estimated by linear regression may be biased (or unstable, due to multicollinearity). More details on describing how to estimate the fusion parameters and the sensitivities may be added.

*We agree that in case of highly correlated views, this might be an issue. Therefore to address this, in the new implementation we are regularizing the meta-model, using ridge regression. Ridge regression is a well established approach to addressing issues of multicollinear or correlated predictors in multiple linear regression. We noted this change in the Results section of the revised manuscript and rerun all experiments using ridge regression to train the meta-model.*

Minor

1. Line 35, G_c=∑_(j=1)^n▒〖exp(-(d_ij^2)/j^2 ) (Y_j ) ̃ 〗 , here d_ij might be d_cj.

*We corrected this in the new formulation of the equation for the generation of the paraview.*

1. The hyper-parameters (e.g., the number of trees and their depths of the random forest models) adopted by MISTy should be specified in the manuscript.

*We added details to the Results section. For each view specific model we used 100 full, unpruned decision trees with (rounded) square root of the number of variables selected at every split.*

1. How the fusion parameters (α_v) were normalized?

The meta-model combines predictions of the different views for the same target. They are first trained with regularization. We added details about the ridge regression and the hyperparameter selection in the Results section. For determining the contribution of the views the fusion parameters (except for the intercept) are normalized such that they sum up to one


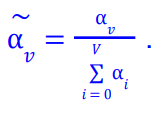


Reviewer #3:

In this work, Tanevski and colleagues propose a method called MISTy which uses spatial transcriptomics data to dissect for each gene or module of genes the contributions of its variation from intra-cellular, local neighborhood and larger neighborhood. For each view MISTy measures the variability captured and the relative contribution of each view for each marker can then be examined. They demonstrate the method using a simulation. They then apply it to a breast cancer dataset in which interactions are actually associated with clinical parameters. An advantage of this method is that it requires no assumptions about that data - for example there is no need for cell type annotation. The method allows for exploration which will be very useful for the community. I think that this method seems to be extremely useful. I am very motivated to test it out.

*We thank the reviewer for the supportive words and positive opinion on our work and for the suggestions, that we attempted to incorporate as follows:*

I do have the following suggestions for the authors to consider:

1. Readability. This will be a difficult read for biologists. Figure 1 is an opportunity to provide an intuition for the method, but it is not well used. The notion of dissecting the sources of variation across the views is a core concept of the paper yet this is not explained by the paper, nor is it well explained in the text. I recommend adding a schematic with examples. Also the notion of feature importance is not well explained in the text. There should be a schematic to also describe this.

*We agree with this, and we now add schematics for different implementations in Supp. Figure 1. We expanded the explanation of feature importance in the “Importance weighting and result aggregation” subsection in the “Methods” section.*

*2*. The simulation is an interesting demonstration of MISTy however the results are discussed only in the text. It would be useful to also establish the expected results for each of the genes shown in Figure 2B and then compare these to the results of the simulation. The authors can establish an expectation that ECM for example would only be detected as capturing variance in the paraview. They can rank the genes and compare with the observed results in the simulation.

*This is a valid point. We extended the in silico model and explained in the text how the ground truth was constructed from the direct interactions of the in silico model. Further, we included Figure 3E, where the importance score of all possible intra- and paraview interactions are shown together with the ground truth interactions.*

It is very interesting that the authors can compare samples according to the variance captured across the three views of the 26 markers (Figure 3 analysis). The authors claim that there is structure in the PCA but they do not quantify this. Also they describe a set of detected markers that contribute most to the PCs - pS6, CAIX, Erk12, and Cytokeratin 7 - but they do not describe what has been previously revealed about these.

*Thank you for pointing out that this information is missing. Our statements are based on the results from the initial publication of this data set (Schapiro et. al. NatMeth 2017), which we were using as our baseline. The initial single cell analysis of this dataset annotated 29 cell types across all samples with various cell phenotypes being present at different frequencies. These cell phenotypes were characterized by specific epitopes as well as combinations of markers. The above-mentioned markers were all found to be linked to more advanced lesions of grade 3 samples in the initial analysis. This includes e.g., hypoxic cells (CAIX+) interacting with active stroma (pS6+/Vimenting+), which aligns with the published single cell analysis. This, our updated analysis recapitulates previous results, but also goes further by showing e.g., luminal cell types (Cytokeratin 7) losing interaction with myoepithelial cells (SMA), which shows that certain structures (ducts / alveoli) are getting lostin comparison to normal tissues. We added more details to the manuscript to help readers with the interpretation of the results.*

4. The authors should also provide a test for whether it is subtypes (rather than grade) of breast cancer that is being captured in the analysis shown in Figure 3E. They could just color by subtypes in the same analysis shown there. Structure according to subtypes would also be interesting.

*We thank the reviewer for this suggestion. We now include a panel with the data from the PCA colored by clinical subtype in Figures 4 and 5 (formerly Figures 3 and 4). We observed that when using the importance signature there is also a visual separation of the samples by clinical subtype.*

5. In the Figure 5 analysis shown in E-G, only three gene pairs are shown. Are these cherry-picked from the full set of interactions? Would other pairs also show a significant change or at least a change in the same direction for survivability?

*Not all estimated importances of the predictor-target pairs are associated with patient survival. We considered the predictor target pairs with significantly correlated importances to overall survival in triple-negative grade 3 tumor samples. In the figure (now Figure 6) we showed the KM curves only for the most correlated predictor-target pair per view. In the revised manuscript this is shown in Supplementary Table 2 where we also added information about the p-value of the log rank test for the KM curves for all pairs.*

6. In Figure 6, the authors detect a relationship between pathways; for example the TNFa and NFkB pathways. Did the authors control for the fact that these pathways may have identical genes? A lack of independence across the gene sets would of course skew the results.

*When modeling the pathway scores with MISTy, we didn’t exclude or control any of the pathways. This was not necessary in this case because PROGENy’s model, that estimates pathway activity scores, was built with a series of independent perturbation experiments, and corrects for the direct overlaps (i.e. the EGFR signature does not include the MAPK signature, and TNF does not include NFkB; Schubert et al Nat Comm 2018). This means that the pathway coefficients aren’t identical. We observed a median Pearson correlation of 0.005 between the footprints of the 14 pathways modeled in MISTy’s application in Figure 7 (previously Figure 6), which in general shows independence between our footprints used to estimate pathway activities. It is true that some parts of the gene expression footprint could agree between pathways in direction, which we would expect t to reflect convergence of the effects on gene expression upon pathway stimulation. Additionally, even though pathways could share a partially similar footprint, the estimated activity will be expectedly distinct given the combinatorial linear combination between the footprint and sparse gene expression. For the purposes of showcasing MISTy, we think that the convergence of TNFa and NFkB footprints is a useful biological case study where intrinsic cellular events are stronger than larger tissue organization events. Thus we kept it on the model since we thought it reflected biology and allowed us to contrast the different spatial contexts modeled with MISTy.*

*Researchers who are willing to follow a similar MISTy pipeline as the one described in the manuscript can choose to perform feature selection based on their prior knowledge or any other condition. In MISTy’s documentation we provided an article that builds flexible pipelines for Visium data that allows for feature and functional view definitions adapted by the user (*[*https://saezlab.github.io/mistyR/articles/mistySeurat.html*](https://saezlab.github.io/mistyR/articles/mistySeurat.html)*).*

7. Figure 6 shows the primary data for Visium but the same is not shown for the breast cancer datasets. Including this in FIgure 3 or 4 could greatly improve the readability of the paper.

*Thank you very much for this suggestion. We added an example image to Figure 4 (previously Figure 3).*

8. Minor comments: In Figure 2B there is an orange circle that is out of place?

*Yes, the circle was misplaced. Thanks for noticing this; we corrected it.*

**Second round of review**

**Reviewer 1**

The authors have addressed most of my previous comments.

Please carefully proofread and edit the manuscript. Some typos or grammatical errors have been noticed:

In the legend of Fig 2E (Page 11 Lines 53-56), “cell types 0 (above) and 2 (below)” should be “”cell types 1 (above) and 3 (below)? Please specify that the importance score is evaluated from the juxtaview.

Page 10 Lines 42-44, “the baseline to compare the multi-view model to” should be “the baseline to compare the multi-view model” ?
Page 23 Line 58, it should be “Supp Fig 6C” rather than “Supp Fig 6D”?

**Reviewer 2**

I am glad to see that the authors have addressed my major concerns. The manuscript has been improved substantially.

An additional comment is that the tissues could be divided into spatial domains based on their spatial context of spots (e.g., https://doi.org/10.1101/2021.08.21.457240). Such spatial contexts and domain borders could be very useful to dissect spatial relationships. The authors could discuss this to clarify their connections.

**Reviewer 3**

The authors have done a professional job in addressing all of my concerns on the original manuscript. Since then, in my lab we have used MISTy and have found it very useful. I completely support the publication of this manuscript and believe that it will be well received by others in the community.
